# Confidence Sharing: An Economic Strategy for Efficient Information Flows in Animal Groups

**DOI:** 10.1371/journal.pcbi.1003862

**Published:** 2014-10-02

**Authors:** Amos Korman, Efrat Greenwald, Ofer Feinerman

**Affiliations:** 1 Laboratoire d'Informatique Algorithmique: Fondements et Applications (LIAFA), CNRS & University Paris Diderot, Paris, France; 2 Department of Physics of Complex Systems, Weizmann Institute of Science, Rehovot, Israel; New York University, United States of America

## Abstract

Social animals may share information to obtain a more complete and accurate picture of their surroundings. However, physical constraints on communication limit the flow of information between interacting individuals in a way that can cause an accumulation of errors and deteriorated collective behaviors. Here, we theoretically study a general model of information sharing within animal groups. We take an algorithmic perspective to identify efficient communication schemes that are, nevertheless, economic in terms of communication, memory and individual internal computation. We present a simple and natural algorithm in which each agent compresses all information it has gathered into a single parameter that represents its confidence in its behavior. Confidence is communicated between agents by means of active signaling. We motivate this model by novel and existing empirical evidences for confidence sharing in animal groups. We rigorously show that this algorithm competes extremely well with the best possible algorithm that operates without any computational constraints. We also show that this algorithm is minimal, in the sense that further reduction in communication may significantly reduce performances. Our proofs rely on the Cramér-Rao bound and on our definition of a Fisher Channel Capacity. We use these concepts to quantify information flows within the group which are then used to obtain lower bounds on collective performance. The abstract nature of our model makes it rigorously solvable and its conclusions highly general. Indeed, our results suggest confidence sharing as a central notion in the context of animal communication.

## Author Summary

Cooperative groups are abundant on all scales of the biological world. Despite much empirical evidence on a wide variety of natural communication schemes, there is still a growing need for rigorous tools to quantify and understand the information flows involved. Here, we borrow techniques from information theory and theoretical distributed computing to study information sharing within animal groups. We consider a group of individuals that integrate personal and social information to obtain improved knowledge of their surroundings. We rigorously show that communication between such individuals can be compressed into simple messages that contain an opinion and a corresponding confidence parameter. While this algorithm is extremely efficient, further reduction in communication capacity may greatly hamper collective performances.

## Introduction

### Background and motivation

Animals living in groups sense their surroundings both directly, by environmental cues, and indirectly, through countless social interactions. There is an abundance of experimental evidence for the usefulness of social information in increasing both the range (the “many eyes” principle) [1]–[5] and the accuracy (the “many wrongs” principle) [6]–[9] at which environmental signals are perceived. Despite these advantages, there are many scenarios in which animals tend to prefer personal knowledge and direct environmental cues to social information [10], [11]. Indeed, second hand information about the environment can become increasingly obsolete [12], [13], distorted [14], and partial [15] as it passes from one individual to the next and, subsequently, lead to maladaptive responses [11]. These contradicting evidences call for a more comprehensive understanding of the usefulness of social information exchange and its limitations under noise.

A distinction can be made between passive and active social messaging [16]. Passive information [17], [18] is transferred as inadvertent cues [19], *i.e.*, with no direct intention of signaling, evident by the behavior of one animal are perceived by others. As an example, models of complex flocking behaviors typically rely exclusively on passive interactions in which animals align their movements to those performed by their neighbors [6], [20]. However, there is evidence that passive information is often accompanied by active, or intentional, signals that communicate part of the animal's internal state. In cooperative situations [21] active signals may enhance the effectiveness of passive cues and lead to faster and more accurate performance [13], [14].

While elaborate active communication has its advantages, simplicity is, nonetheless, important. Indeed, it is required that communication remain energetically cheap [22], cognitively manageable [23], [24] and concise [21]. A main goal of this work is to identify *simple* active communication schemes that enhance the reliability and the benefits of social information.

Animal groups, together with numerous other biological ensembles, are naturally described as entities that collect, share and process information. Unfortunately, with the exception of neuroscience [25], the potential of information theory in providing rigorous descriptions of such ensembles remains, largely, unrealized [15]. For example, the term “information flow” is often used to describe the gradual process in which messages are being relayed between agents [26], [27]. Although the speeds and directionality of information flows have been described for several systems [1], [28]–[30], it remains unclear how to rigorously analyze such flows to quantify the amount of transferred information. A second goal of this paper lies in introducing information theoretical tools as a means of quantifying information flows within a group of agents.

In what follows, we use an algorithmic perspective [31]–[33] to tackle the question of information sharing within a population of cooperative agents. The agents use environmental cues intertwined with social interactions to obtain ever refined estimates of some fixed, unknown environmental target value [34]. Interactions include both passive and active components. A passive observation amounts to obtaining a noisy measurement of the observed agent's behavior. An active signal exposes some part of the observed agent's internal state. We are interested in how active signals may be economically used to best enhance the flow and benefits of passive communication.

To study this question we compare two models. The *non-restrictive* model allows for infinite resources in terms of memory, active communication and individual computation. On the other hand, the *compact* model restricts active communication and memory to a single parameter and individual computation to a constant number of the basic arithmetic operations. We present recent experimental observations [14], [35]–[37] as well as novel evidence regarding ant interactions that suggest that the communication of a self-confidence parameter is a relevant process within animal populations. Inspired by such observations, we propose a simple and natural algorithm for the compact model that relies on the sharing of confidence. This model can serve as a basic representative of the family of confidence-sharing algorithms. We show that the performances of this algorithm are highly competitive with those of the best possible algorithm for the non-restrictive case.

One may be tempted to reduce active communication below what is permitted by the compact model, but we show that this may incur a heavy price in performance.

### The model

#### Formulation of the problem

We study a simple model for the sharing and dissemination of information within a population of anonymous agents (see section 1 in Text S1). Each agent, 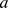, is associated with an external state 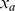 which represents, for example, its physical location or direction of motion. The goal of each agent (following [34]) is to modify this state so as to be as close as possible to a target value, 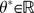. More formally, for each agent 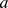, we view its external state 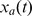 at time 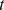 as an *estimator* of 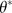. At any given time, the agent may modify its external state such that it is maintained as an unbiased estimator with minimal mean square error (MSE). We stress here that this work is restricted to this specific cost function and that other estimators require further study (see, for example, [7], [37], [38]). For the sake of conciseness, from here onwards, we refer to 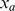 as “location” and to a change in 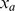 as a “move”.

To initialize the system, the location 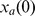 of each agent 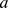 is randomly chosen according to some arbitrary distribution 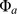 centered at 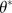. We assume that the variance of 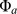 is known to agent 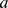. The agent may store this and other pieces of information it collects in its *memory*.

Agents improve their estimation of 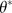 by relying on both social interactions and environmental cues, where in-between such events they are free to perform moves and adjust their memory state. Technically, environmental cues are included by having a particular, immobile set of agents represent the environment. For simplicity of notation, we focus on pair-wise interactions which can be either uni- or bi-directional (our results transfer to interactions that involve a larger number of agents in a straightforward manner). The information transferred in such interactions may contain both active and passive signals. Passive information is obtained as agent 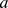 measures its current relative distance [39] from agent 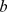, that is, 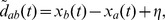
where the additive noise term, 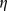, is chosen from some arbitrary distribution 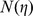 whose variance, 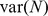, is known to the agents. Active signals are modeled as messages that expose some part of the internal memory of agent 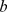 to the observing agent 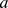.

#### A remark regarding related problems in other disciplines

The problem we address is somewhat related to the *Relative Location Estimation* problem studied within the context of sensor networks [39]. There are, however, important differences of emphasis between these two cases. First, most sensor localization algorithms are designed for static sensors [39] and are often, to some extent, centralized [40]. Our setting is inherently distributed and moreover, mobile; agents continuously update their location in a way that effects subsequent distance measurement by others. Second, restrictions on internal memory and computation of sensors are typically not as constraining as those we consider here (especially in the case of actively mobile sensors [40]). Finally, while sensor localization algorithms typically focus on triangulation solutions that rely on fixed communication networks with unique identities [41], our setting is anonymous and does not allow agents to control with whom they interact. The question we face is further related to computer science problems such as *consensus* and *gossip* [42], however these are typically discrete in nature, and do not take communication noise into account.

## Results

### The optimal algorithm

To evaluate the performances of algorithms, we compare them to Opt (see section 2 in Text S1), the best possible algorithm operating under the non-restrictive model.

Being as liberal as possible, we further assume that active communication is completely reliable. This is since any definition of active noise must depend on a particular choice of a communication scheme which, in turn, may restrict an optimal algorithm. Moreover, here, agents are initially provided not only with the variances of the noise and initial distributions but also with their full functional forms. That is, the memory of an agent 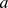 initially contains 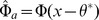 and 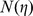. Without loss of generality, the memory of an agent further includes a vector that contains all prior moves and distance measurements it took. Following an interaction, the observing agent adds to its memory not only the new noisy distance measurement but also the full memory content of the observed agent. This leads to the accumulation of large nested data-structures. The agent may then perform arbitrarily sophisticated computations over its memory to adjust its location 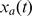 to its best possible estimate of 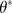.

We stress that none of the proofs in this manuscript rely on the identification of an optimal algorithm. Nevertheless, for the sake of completeness, we specify Opt for independent meeting patterns (section 1.1.3 in Text S1), which are especially meaningful on short timescales or if the system is highly mixed. Indeed, in such cases, algorithm Opt can be precisely described (section 2.2 in Text S1). Specifically, each agent maintains a 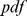 that represents the relative positioning of the target value 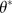 with respect to its current location. The pdf is initialized to be 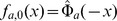. Upon observing another agent 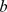 at time 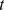, agent 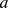 performs the following operations:

**Algorithm Opt**

- **Compute:** normalize the next integral to obtain a 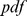 (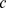 is a normalization constant):

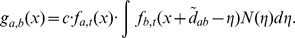

- **Update external state:** 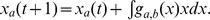

- **Update memory:** 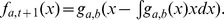

In general, as time passes, the description of the stored 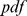 requires an increasing number of moments and its communication a more elaborate encoding scheme. Moreover, the calculations required for updates become increasingly complex.

### Difficulties towards efficient information fusion

Algorithm Opt relies on the transmission and updates of probability functions and on relatively complex calculations. We wish to identify a simple algorithm whose performance is highly competitive with that of Opt. To do this one faces several difficulties.

A first difficulty lies in the fact that the partial knowledge held by each agent is relative (*e.g.*, an estimation to the distance between this agent and 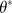) and hence may require the agents to carefully fuse other perspectives than their own. This difficulty is enhanced, as the agents are constantly on the move. We have shown how *non-restrictive* algorithms may overcome such difficulties if each agent encodes all its previous moves in memory and then uses this information to deduce absolute measurements (section 2.1 in Text S1). In compact models, such tactics lose their effectiveness and it is not clear how agent 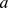 should treat distance measurements to an agent 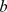 whose position constantly changes over time.

**Figure 1:**
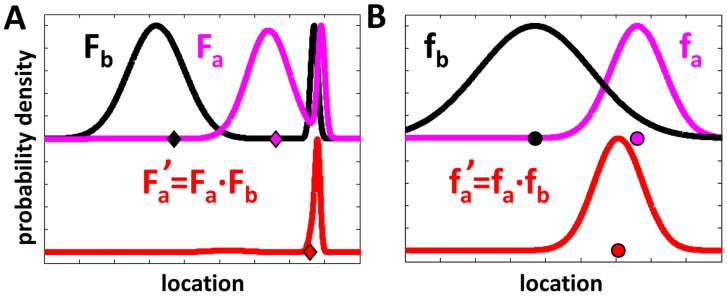
Storing and communicating detailed assessments of target location is, in some cases, extremely beneficial. **A.** The memories of (pink) agent 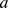 and (black) agent 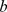 are represented by a 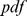 (capital F's) that summarizes their full information regarding the target. The agents locate themselves at the mean of their corresponding 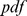 (marked by filled diamonds). We consider an interaction in which agent 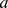 observes agent 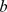 and updates its position and memory to those depicted in red. **B.** The agents are initiated as in A. However, before they interact, their memories are compressed into Gaussians (lowercase f's) that agree in mean and variance with their previous assessments. Note that since the mean values have not changed, the initial locations of the agents in both panels are identical. Following the interaction, agent 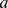 has moved to a different location (compare red diamond to red circle) and has gained less information (compare variances of red curves) when compared to the case in which compression had been avoided. For simplicity, all interactions in this figure were taken to be noiseless.

It is known that a reasonable way to combine estimators is to form linear combinations in which each estimator is weighed by its inverse variance [43]. Although this is the best estimator that could be formed as a linear combination it is not overall optimal. Indeed, maintaining and communicating highly detailed memories can, in some cases, significantly improve an agent's assessment of the target value (for example, see Figure 1).

This problem worsens in the context of an interacting population. Here, maintaining a high degree of detail requires storing an arbitrary number of 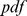 moments which may grow with every interaction. Discarding this accumulating information by repeatedly using simple (*e.g.* linear) estimators could, therefore, lead to performances that deteriorate with time. Hence, it is not clear how to compress the information held by agents into few meaningful parameters while avoiding the accumulation of errors and runaway behavior.

Another of the analysis difficulties corresponds to the fact that the 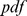 held by an agent at time 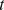 depends on many previous deviation measurements in a non-trivial way, and hence the variance of a realization of the 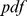 does not necessarily correspond to the variance of the agents' opinion, when taking into account all possible realizations of all measurements. Hence, one must regard each 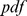 as a multi-variable distribution. A further problem has to do with dependencies. The independent meeting pattern guarantees that the memory 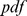
's of two interacting agents are independent, yet, given the 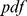 of the observing agent, the 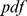 of the observed agent and the deviation measurement become dependent. Such dependencies make it difficult to track the evolution of an agent's accuracy of estimation over time. Indeed, to tackle this issue, we had to extend the Fisher information inequality [44], [45] to a multi-variable dependent convolution case.

### The biological relevance of confidence based algorithms

Internal representations of confidence have been shown to affect animal behavior over a wide range of species [46]–[49]. Confidence as an internal parameter that builds up as a passive agent gathers external evidence has been measured in pre-decision neuronal responses (see, for example, [50]). The notion of confidence as an internal parameter carries over into group contexts wherein animals were demonstrated to become more responsive to social information as their own certainty drops [37], [51], [52].

Furthermore, evidence also suggests that animals are capable of communicating their confidence as well as assessing that of their conspecifics [13], [14], [35], [53]. One such example comes in the context of conflict, where threat behaviors may indicate the communication of confidence. While no single work directly binds all elements of confidence sharing many supportive evidences exist: Dominance hierarchies, like confidence, are constructed according to the accumulation of evidence [54]. Further, threats are correlated with large differences in dominance rank [55] and are often non-deceptive [56]–[58] and convey the animal's actual chances of winning the next fight. Moreover, threats are generated and perceived at different levels of intensity [55], [59] to the extent of causing an opponent to back away from confrontation [53], [60].

Other examples come from more cooperative scenarios such as house hunting behavior in honeybees (*Apis mellifera*). It was shown that swarming bees collectively move towards a new nest site by communicating two-component messages: The direction in which bees fly encodes the desired direction towards the new site while the speed of flight determines the degree of responsiveness this message will elicit in others [61], [62]. Furthermore, it was shown that high speed is associated with bees that have been to the new site (streakers) as well as bees that do not have first hand accounts but whose flight is generally directed towards the desired site [61]. These evidences are consistent with an analogy between flight speed and confidence regarding the correct direction to the new site. Another example occurs earlier in the house-hunting process. The messages which scouts convey regarding the location of prospect nest sites contain (at least) two components: While the direction to the advertised site is encoded by the waggle dance, the intensity of the message is encoded in the number of times the bee performs this dance [63], [64]. The intensity of the message correlates with the quality of the advertised site and could be interpreted as the confidence of the bee that the site she advertises is the best of all options. This interpretation is strengthened if, similar to what has been shown for ants [65], [66], bees have some internal scale to the quality of a site.

**Figure 2:**
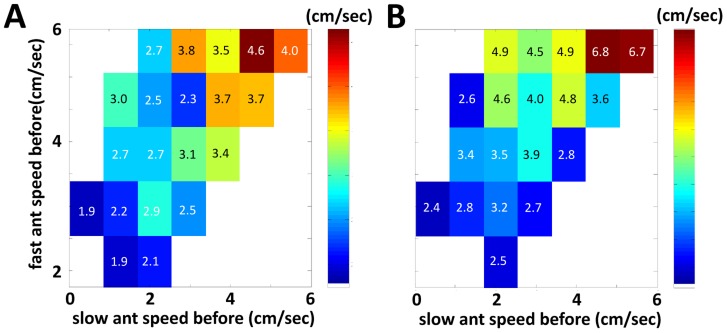
*C. niger* recruitment behavior exhibits interactions that resemble Conf. The figure summarized the speed change of ants directly before and after an interaction within the nest. We refer to the two interacting ants as the fast/slow ant according to their speed before the interaction. Identifying speed with confidence about the presence of a food source [14] reveals an interaction rule similar to that suggested by Conf. **A.** Mean speed of the slow ant following an interaction. **B.** Mean speed of the fast ant following an interaction. The figure summarizes (n = 429) interactions and demonstrates how the speed of an ant after an interaction increases as either her prior speed or the prior speed of the ant she interacts with are larger. For example, we find that the mean speed at which an initially slow ant (
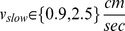
) exits an interaction with a relatively fast faster ant (
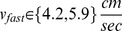
) is higher than the her speed after an interaction with a relatively slow faster ant (
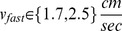
), 
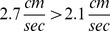
, 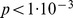 by the one-sided two-sample Kolmogorov-Smironov test. Similarly, the mean speed of a fast ant (
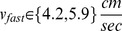
) increases more after encountering a relatively fast slower ant (
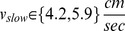
) than a relatively slow slower ant (
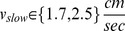
): 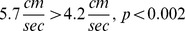. Using the same speed ranges we also find that the mean speed of a slow ant after an interaction is an increasing function of her speed prior to the meeting: 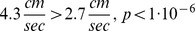 and that the same holds for fast ants: 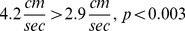. For further details regarding a general slow-down in speed evident after each interactions see [14].

A further example for the role of confidence during interactions comes from recruitment behavior in the desert ant *Cataglyphis niger* [14]. Here, ants within the nest interact with their nest-mates to accumulate indirect evidence regarding the presence of food and towards an active decision to exit themselves (recruitment). Similar to the accumulation of neuronal activity that proceeds a decision [50], ants were observed to gradually increase their speed of movement before deciding to exit the nest [14]. Furthermore, ants which have been in direct contact with the food are certain of its presence and indeed maintain high speeds for extended periods of time [14]. These evidences suggest that an analogy between the speed of an ant and her confidence may be useful. In Figure 2 we present novel empirical evidence of the way ants update their speed following an interaction. This data confirms that speed (confidence under this analogy) is both transmitted and perceived by the ants. Moreover, the speed of an ant after the interaction is an increasing function of both her speed and the speed of her interacting partner prior to the interaction.

### A basic confidence-based algorithm

Having identified confidence sharing as a relevant communication scheme in animal groups, we turn to study the compact algorithm Conf: a *basic* representative of the family of algorithms that rely on the active communication of confidence. This algorithm is basic in being both simple and natural: It is simple as it is highly economical in terms of communication, memory usage and internal computations. It is natural since it relies on linear combination information fusing techniques. Below, we describe Conf and show that it displays near optimal performance.

In algorithm Conf each agent, 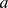, stores in its memory a single parameter 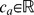 that represents its *confidence* regarding its current distance from the target 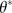. The initial confidence of agent 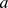 is set to 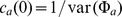. When agent 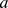 observes agent 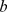, it receives both the passive noisy distance measurement 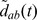 and an active message containing the confidence parameter of 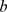. This information will then allow agent 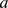 to relocate itself by using a *weighted average* procedure [34], [43]. Then, a suitable update is made for 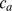 to reflect 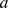
's confidence of its updated location.

Specifically, upon receiving 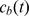 and 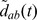, agent 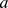 proceeds as follows:

**Algorithm Conf**

- **Compute:** 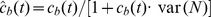.
- **Update external state:** 
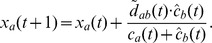

- **Update confidence:** 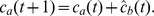

### Competitive analysis

We provide a rigorous proof (section 5 in Text S1) that the performances of Conf are very close to those of Opt when the meeting patterns are independent and active communication is noiseless. Specifically, we first show (section 5.1 in Text S1) that under these conditions, the rules of Conf guarantee that the location 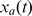 of any agent 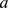 serves as an unbiased estimator of 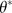 and that the confidence 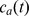 satisfies: 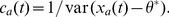
(1)


**Figure 3:**
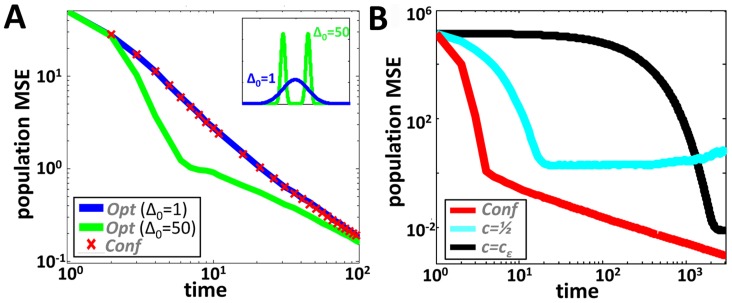
Comparing the performances of the confidence based algorithm, Conf, to those of other algorithms. **A.** Optimal algorithms. We look at the convergence of an optimal algorithm for two different initial distributions. Double Gaussian initial conditions (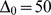) possess higher Fisher information than Gaussian initial conditions with the same variance (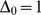) and thus converge faster. As the Conf algorithm uses only variances it performs equally well under the two conditions. Note that for Gaussian initial conditions, Conf is optimal while, for the double Gaussian case, the competitiveness of Conf is, at any time, much smaller than the theoretically predicted upper bound of 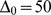. **B.** When compared to Conf, linear combination algorithms exhibit a large deterioration in performances and a speed-accuracy tradeoff: The simple average algorithm (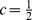) converges relatively fast but to a steady state that is dominated by the amplitude of the communication noise. The linear combination algorithm with 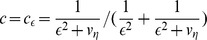 (where 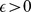 can be taken to be arbitrarily small) reaches a tight steady state at the cost of long convergence time.

We further show (section 5.2 in Text S1) that although approximation errors that result from the information compression of Conf are inevitable, they do not accumulate with time and through repeated interactions. Indeed, the quotient between the variance of the population under Conf and its variance under Opt remains bounded, at all times, by the initial Fisher-deviation 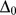 (as defined in the Materials and Methods). More specifically, under algorithm Conf, the variance of any agent 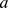 at time 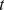 is bounded by 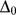 times the corresponding variance under Opt (see Figure 3A):
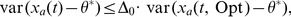
(2)where 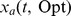 denotes the location of agent 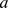 at time 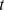 under algorithm Opt.

To prove Equation 2, we relate the variance of Opt to a measure of information which we call the *relative Fisher information*. This measure, denoted 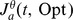 (formally defined in Text S1, section 3.2), quantifies the agent's current knowledge regarding 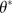. Intuitively speaking, this notion can be thought of as the Fisher information of the 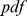 family that describes the random samples held by 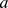 under algorithm Opt with respect to the translational parameter 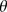 (see Materials and Methods). We then use the Cramér-Rao bound to deduce that: 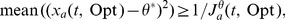
(3)where the mean is taken over all possible random initial locations and communication noises, as well as, possibly, over all random choices made by the agents themselves.

We then show that the confidence of an agent 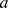 under algorithm Conf satisfies: 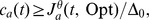 which establishes Equation 2 and proves that the competitiveness of Conf with respect to Opt is, at most, the initial Fisher deviation 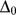.

Note that for 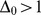, the optimal algorithm Opt cannot, in fact, achieve the Cramér-Rao bound at all times (t = 0 being a trivial example). Therefore the competitiveness of Conf with respect to Opt can be expected to be even tighter than 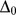. This is indeed verified by simulation (see Figure 3A). Moreover, we show that in the case of Gaussian noise, and regardless of 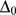, the performance of Conf will approach that of Opt at large times (section 5.2.3 in Text S1 and Figure 3A). Note that in the case in which the noise and initial distributions are all Gaussian, the Fisher deviation satisfies 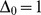 so that Conf is optimal (Figure 3A).

### Algorithms without active communication

We next compare the Conf algorithm to even simpler algorithms that rely solely on passive communication.

We first consider algorithms in which the interaction update rule is a simple linear combination of the observing agent's location, and the estimated location of the observed agent: 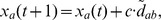
for some constant 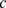 (note that in algorithm Conf, 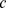 is not constant and is set according to the active message and 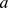
's current confidence). A simple average algorithm is obtained by setting 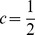.

The performance of constant linear combination algorithms is of interest since they require minimal resources: agents are not required to store any memory of their current internal state. We find that, in general, when communication noise is substantial, linear combination algorithms do not perform well. They exhibit a speed accuracy tradeoff converging within a time scale of 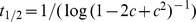 (which diverges for small values of 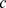) to a steady state with a variance that scales as 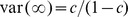 (section 7 in Text S1 and Figure 3B). On the other hand, in the case of uniformly informed populations and negligible communication noise, the performances of the simple average algorithm (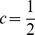) approach those of Conf in terms of both convergence rate and steady state variance. Intuitively, simple averaging functions well under these circumstances since the information held by two interacting agents, at any time, is of equal quality.

Active communication can also become redundant when passive communication noise is very large with respect to the uncertainty of the agents. Indeed, in this case, a “passive” algorithm is obtained by translating the rules Conf into a high noise regime. The effective confidence of any observed agent becomes 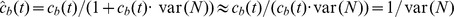 which is independent of its actual internal state.

Conversely, “passive” algorithms are expected to fail in situations where noise levels are comparable to agent uncertainty and knowledge is non-uniformity distributed among the agents. In this case, the assumption that an observed agent's confidence is 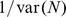 fails and could lead to irreparable mistakes in the observing agent's position and confidence after the interaction. For intuition, consider the extreme case in which a single agent has very accurate knowledge of the target value while all other agents have no information at all. In this case, Conf would allow for very fast convergence typical of rumor spread: roughly within 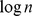 rounds, where 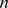 is the number of agents. On the other hand, if no active communication is allowed, it becomes difficult to distinguish the knowledgeable agent within a large population of anonymous agents (see section 7.1 in Text S1).

### Generalizations

#### Information flows, Fisher capacity and convergence times

We now set to find lower bounds on the convergence time of a group of agents applying an arbitrary algorithm. First, we note that an agent's relative Fisher information, 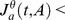, remains well-defined with respect to any algorithm 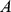, any interaction pattern and any noise in either active or passive communication (section 3 in Text S1). An inequality similar to that formulated for Opt (equation 3) also holds for this, more general, case: 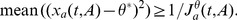
(4)


Next, we combine equation 4 and a bound on the relative Fisher information an agent can gain through an interaction to produce lower bounds on collective convergence times.

**Figure 4:**
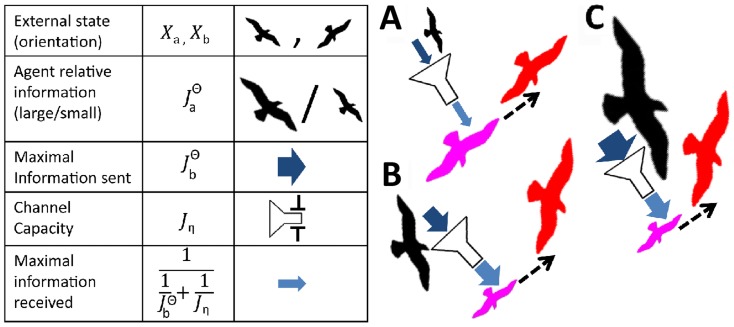
Information flow. The table on the left hand side relates the terms used, their notations in the text, and their graphic representation per panels A–C. In A–C the pink agent observes the black agent and updates its state (dashed arrow) to be the one depicted by red. **A.** An agent with high Fisher information effectively ignores one with less knowledge. **B.** A weighted average. Following this interaction, the observing agent updates its orientation and increases its Fisher information. **C.** Although the information held by the black agent is much higher than in panel B, its effect does not grow. This is a consequence of the restricted Fisher Channel Capacity.



By generalizing the Fisher information inequality [44], [45], we prove (section 4 in Text S1) that, under an independent meeting pattern, when agent 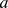 observes agent 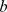 at time 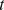 then: 
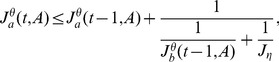
(5)where 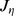 is the Fisher Information of the noise distribution family 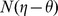 (see, also, Figure 4) In particular, this implies that:

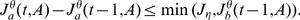
(6)


Intuitively speaking, agent 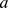 cannot obtain more information than that stored in 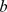 or a measurement more precise than allowed by communication noise. This equation holds with respect to any level of noise in active communication, and in particular, when active communication is noiseless. The bound of 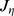 on information increase holds with respect to any algorithm 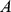, hence, we can view it as a property of the information channel itself. In analogy to Channel Capacity as defined by Shannon [67] we term 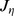 as the *Fisher Channel Capacity*. This definition is not to be confused with the notion of “Channel Capacity” previously used by Frieden *et al.*, in a different, non-distributed context. Figure 4 illustrates and summarizes the above stated ideas.

These restrictions on information flow can be translated into lower bounds for convergence times, *i.e.* the time in takes the whole population of agents to enter a certain tight window 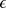 around 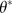. Convergence requires that the estimator applied by a typical agent have a variance that is on the order of 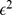. As this variance must comply with the Cramér-Rao bound, the Fisher information of the typical agent in the system has to exceed 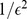.

To get some intuition on the convergence time, let 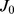 denote the median initial Fisher information of an agent (this is the median Fisher information over the distributions 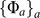), and assume 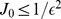. Equation 6 implies (section 6 in Text S1) a bound for the best possible convergence time 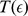: 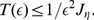
(7)


Let 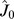 denote the maximal initial Fisher information over all agents. In the case where 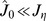, one can obtain a tighter upper bound for 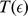. Note that the Fisher information at an agent is always at most the corresponding Fisher information in the analogue scenario where there is no noise in both passive and active communication. For this noiseless scenario, the additive property of Fisher information implies that the maximum Fisher information over all agents grows by, at most, a factor of 2 in each round. This leads to the following bound: 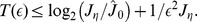



#### Dependent interaction patterns

**Figure 5:**
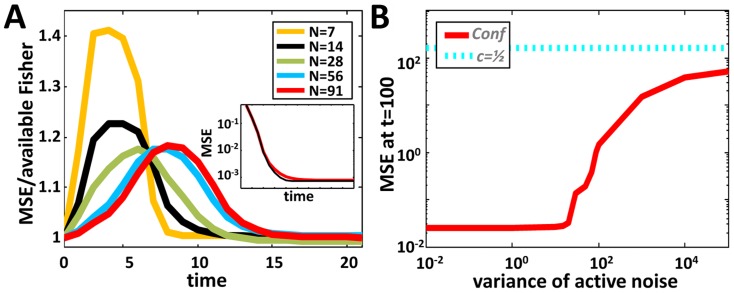
Performance of Conf under general conditions. **A.** Dependent interaction patterns. Quality of the convergence for small populations as depicted by the population variance normalized by the optimal variance allowed by the Cramér-Rao bound. A ratio of 1 implies optimality. The inset shows the MSE of Conf (red curve) and lower bound (black curve) for the case 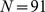. **B.** Convergence performance of Conf for different levels of noise in the active communication. The x-axis specifies the variance of the random Gaussian term that multiplies confidence transmissions. The dashed line signifies the performance of a simple-average algorithm.

Our proofs pertain to an independent interaction regime which, in the strict sense, restricts our analysis to highly connected interaction graphs or short enough times. We used simulations to test the effectiveness of Conf on small populations that may better capture a natural settings where interactions are spatially constrained. This was done by comparing the MSE achieved by an agent employing Conf to the reciprocal of the Fisher information of this agent's under algorithm Opt. For simplicity, we considered a noiseless scenario; this allowed us to precisely calculate the corresponding Fisher information. We found that, on average, algorithm Conf remains extremely efficient for dependent meeting patterns that result from small population sizes. Deviations from optimality are both extremely small and transient (Figure 5A).

#### Noisy transmissions of confidence

The continuous nature of algorithm Conf suggests that it may also be robust under noise in confidence transmission. We therefore used simulations to test the effects of noisy active communication. Noise was realized as a multiplicative Gaussian term to maintain the non-negativity of confidence. Figure 5B agrees with our hypothesis showing that Conf is highly robust to communication noise. Note that for all levels of noise, Conf still outperforms the simple average algorithm. The robustness of Conf further implies that the transmission of confidence is not required to be analog but could be binned into a restricted number of bits.

#### Dynamic environments

We have proven that algorithm Conf is highly competitive in static environments. However, it cannot be expected to perform well in dynamic environments. This is due, for example, to an erroneous buildup in confidence amongst interacting agents with similar opinions [13]. In this case, agents may ignore subsequent environmental changes due to over-confidence. Alternative, more complex algorithms that rectify this phenomenon have previously been suggested [7].

To resolve such issues, we present two extensions of Conf (see section 8 in Text S1). The first algorithm, fully described in Text S1 section 8.1, relies on a single extra bit that is stored in the agents' memory and is turned “on” if an agent is updated. The agents are further required to be able to measure time in a way that allows them set this extra bit to zero when their information becomes obsolete. In figure S1A, we show that while this algorithm coincides with Conf in periods where the environment is stable it also allows very rapid population shifts that track a sudden change in environmental conditions.

A second algorithm relies on a weighted-average rule that is corrected for non-independent observations (see Oruç *et al.* [68]). This algorithm assures, for example, that confidence will only marginally increase following an interaction between agents with highly correlated information. We ran a simulation that uses this rule in an interacting population of uniformly informed agents. Indeed, we found that at steady state, the Fisher information in each agent exactly equals the initial Fisher information of the entire population (see section 8.2 in Text S1 and figure S1B). In other words, all initial information has disseminated between the agents while over-confidence has been avoided. Since the agents are not over confident, they remain responsive to environmental changes which they quickly track (see section 8.2 in Text S1 and figure S1C). Thus, this algorithm improves on the flexibility of Conf. However, this extended algorithm cannot function, as is, in non-uniform populations as two interacting agents have no simple method for assessing the correlation between their estimates prior to an interaction.

#### Heterogeneous populations

Experiments have demonstrated how, when two humans make a joint decision, they weigh their opinions not by the variance of their uncertainty (as could be expected for optimality) but by its standard-deviation [69]. A possible explanation for this was suggested by Ernst [70] who noted that dividing a measurement by its standard deviation yields a unit-less quantity that may facilitate communication between people who may differ in their perception of distance or happen to be using different units of measurement.

As differences in perception are also bound to occur in animal populations it is interesting to test how Conf, which uses inverse-variance weights, performs in this setting. For this, we simulated heterogeneous populations in which each individual perceives distance differently, for example a 1.5-biased individual will measure a distance to be 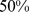 larger than it actually is while a 1/3-biased individuals will perceive distances to be smaller by a factor of 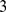. Simulating algorithm Conf on such populations, we found (see section 9 in Text S1 and figure S2) that it continues to perform well in populations with a perception heterogeneity that goes as high as a factor of 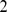 (implying differences of up to a factor of 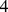 between the perception of different individuals). When biases bypass the order of the signal itself Conf starts to lose its absolute advantage over an algorithm that does not communicate confidence at all.

## Discussion

In this work we theoretically studied an abstract model of animal communication within a group which generalizes the work of McNamara and Houston [34]. Similar to their approach, we considered a basic model which enabled us to perform rigorous analysis, often impossible in more complex scenarios. We have shown that weighted averaging algorithms, previously known to be efficient for fusing multiple pieces of evidence [43], naturally carry over to a scenario in which a group of agents share and aggregate information. The weights used may be interpreted as the agents' confidence in their opinion.

We have theoretically shown, that, remembering and *actively* communicating confidence is, in fact, sufficient for near-optimal decisions in cooperative group contexts. Using the confidence measure is straightforward: individuals with high confidence are more persuasive while those with low confidence more fickle. Finally, the fundamental nature of our model makes our results potentially relevant to a large number of natural systems.

We have used the framework of Fisher information to study information flows within cooperative groups. In particular, we have defined the *Fisher Channel Capacity* and demonstrated how it bounds collective reaction times. This opens the door for further rigorous quantifications of information flows within animal groups.

We introduced Conf, a simple weighted-average based algorithm that uses compact memory and communication in a way that overcomes the anticipated shortcomings of information compression (*e.g.*, see Figure 1). We have shown that Conf is highly competitive when compared to an optimal algorithm that utilizes maximal memory, communication capacity, and computational resources. In fact, we bound the difference in performance by a constant factor - the *initial Fisher-deviation*.

We have presented evidence that supports the relevance of Conf to actual biological groups and turn to suggest how this may be helpful for analyzing experimental data. A most intriguing result would be to utilize Equation 7 to obtain a lower bound on communication noise levels. Indeed, Equation 7 holds with respect to any algorithm operating in the corresponding setting, and with respect to any level of noise in active communication. If the setting is matched in an experiment, the initial variance is large, and the convergence time fast, Equation 7 would yield a lower bound on 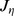, the Fisher information in the noise corresponding to the passive communication. Such a result would demonstrate the usefulness of the indirect methodology, based on algorithmic lower bounds as suggested in [71]. Moreover, such a lower bound on the amount of noise seems to be difficult to obtain by other, more direct, methodologies.

Further practical implications of our results include the identification of scenarios in which active communication is likely to be employed. These include cases in which the noise level is intermediate and situations of populations that are variable in terms of initial knowledge as is the case in effective leadership scenarios [36], [38]. In such cases, our results suggest that it may be useful to search for the active transmission of “confidence” signals, which can be encoded *e.g.*, in the speed of agents [14], [61].

Our analysis for the performances of Conf assumes independent meeting patterns. Such patterns are especially meaningful when agents rely on few interactions each, or when the system is highly mixed. We have used simulation to demonstrate that algorithm Conf continues to perform well for small groups in which interaction patterns are no longer independent. In addition, our simulations show that Conf is robust under active communication noise, heterogenic populations, and that simple extensions of this algorithm may be expected to perform well in dynamic environments.

It is interesting to identify those scenarios in which active communication appears to be of lesser importance. When personal information is reliable and frequently updated there is, trivially, no requirement for any sort of communication. It is when personal information is less accurate that social information becomes useful. We have shown that simple averaging algorithms (operating without long term memory) behave well in uniform populations with communication noise that is negligible in comparison to the desired convergence state. We further showed that when communication noise is very large then an algorithm in which each agent maintains an internal confidence measure but does not communicate it [38], [72] performs extremely well. This implies that in such cases, the system can perform well without resorting to active communication.

Although our results were formulated in the language of animal group behavior they can readily be generalized to a large range of cooperative biological ensembles. For example, bacterial quorum sensing is mediated by both passive cues (*e.g.* one cell senses another's waste products) and active signaling mediated by designated quorum-sensing molecules [73].

## Materials and Methods

### Fisher information, and the Cramér-Rao bound

We consider parameterized probability density function (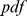) families 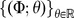 where 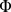 is the functional form and 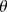 is a translation parameter [45]. The Fisher information of a 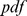 family is defined as: 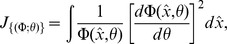
where 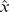 denotes all variables on which 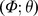 depends. Note, that since 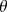 is a translational parameter, the Fisher information is both unique (there is no freedom in choosing the parametrization) and independent of 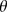 [45]. The Cramér-Rao inequality sets a lower bound on the variance of any unbiased estimator, 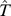 based on a random sample taken from 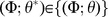, for the parameter 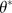: 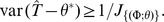



### Initial Fisher-deviation

To define the initial Fisher-deviation, denoted 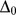, we first define the *Fisher-deviation* of a distribution 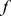 as 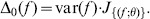



Note that, by the Cramér-Rao bound, 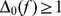 for any unbiased distribution 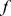.

The *initial Fisher-deviation*  is the supremum of the Fisher-deviations over all the (unbiased) distributions involved, namely, the 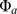 distributions governing the initial locations and the noise distribution 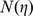. Specifically, let 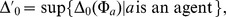
and finally define 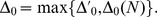



Observe that if the distributions 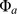 and 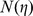 are all Gaussians then 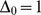.

## Supporting Information

Figure S1 **Extensions of Conf to dynamic environments.**
(TIF)

Figure S2 **Algorithm Conf in heterogenic populations.**
(TIF)

Text S1 **Additional definitions, proofs, and simulations.**
(PDF)
