# Supplementary figures and images for: Confidence Sharing: An Economic Strategy for Efficient Information Flows in Animal Groups
Source: PLoS Comput Biol. 2014 Oct 2;10(10):e1003862. doi: 10.1371/journal.pcbi.1003862 (PMC4183420; doi:10.1371/journal.pcbi.1003862)

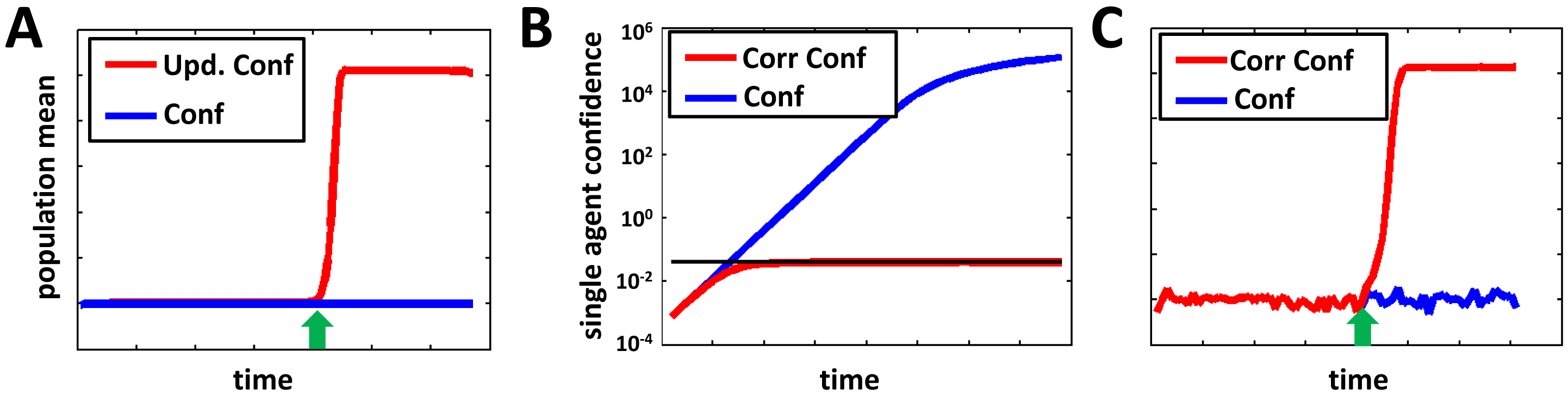

Supplement: Figure S1 — Extensions of Conf to dynamic environments. (TIF) [file pcbi.1003862.s001.tif]

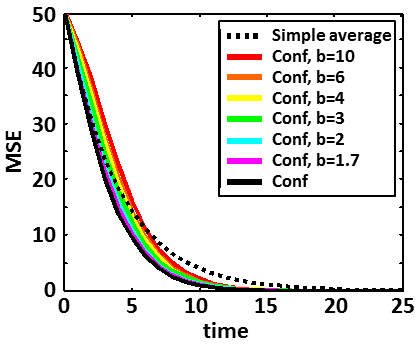

Supplement: Figure S2 — Algorithm Conf in heterogenic populations. (TIF) [file pcbi.1003862.s002.tif]
