# Supplementary material for: Confidence Sharing: An Economic Strategy for Efficient Information Flows in Animal Groups
Source: PLoS Comput Biol. 2014 Oct 2;10(10):e1003862. doi: 10.1371/journal.pcbi.1003862 (PMC4183420; doi:10.1371/journal.pcbi.1003862)
Supplement: Text S1 — Additional definitions, proofs, and simulations. (PDF) [file pcbi.1003862.s003.pdf]

Supporting Information

Confidence sharing: an economic strategy for efficient information flows in animal groups

Contents

1 Preliminaries **3**

1.1 Model definitions 3

1.1.1 Communication 3

1.1.2 Rounds 4

1.1.3 Meeting patterns 4

1.1.4 Random variables 4

2 The optimal algorithm Opt **5**

2.1 Specification of **Opt** 5

2.2 Specification of **Opt** in an independent meeting pattern 6

3 Fisher information and Relative Fisher information **7**

3.1 Fisher information 7

3.1.1 Fisher information and the Cremér-Rao bound 7

3.1.2 Initial Fisher-deviation 8

3.2 Relative Fisher Information 8

4 Upper bounds on the Fisher information $J_a(t, A)$ **10**

4.1 Proof for upper bounds 10

4.2 Extending the Fisher inequality 12

5 An almost optimal compressed algorithm **14**

5.1 Mean and variance of estimators in algorithm **Conf** 14

5.2 Competitive analysis of **Conf** 15
5.2.1 Competitive analysis - definition 15
5.2.2 The competitiveness of **Conf** 15
5.2.3 On the performances of **Conf** at large times 17

6 The Fisher Channel Capacity and convergence times 18
6.1 The Fisher Channel Capacity 18
6.2 Bounds on the convergences time 18

7 No active communication 20
7.1 Passive algorithms 20
7.2 Fixed linear combination of locations 20
7.2.1 Speed vs. accuracy tradeoff in uniform initial conditions 21
7.2.2 The simple average algorithm 21
7.2.3 Obtaining lower variance at the price of long convergence times 21

8 Extensions of Conf to dynamic environments 22
8.1 Algorithm 1: Communicating an extra 'updated' bit 22
8.2 Algorithm 2: Correcting interactions for correlations 23

9 Heterogeneous populations 24

10 References 25

1 Preliminaries

1.1 Model definitions

We study a simple model for information sharing and dissemination within a population of agents. By executing an algorithm A, at any given time t, each agent a holds an external real variable $x_a(t, A) \in \mathbb{R}$ (representing, *e.g.,* its physical location) and possesses some internal state $y_a(t, A)$, which we call *memory.* To simplify the presentation, whenever the algorithm A is clear from the context, we may omit its description, writing, *e.g.,* $X_a(t)$ instead of $X_a(t, A)$. In addition, we term the external state $x_a(t)$ as *location* from now onwards. Initially, the location $x_a(0)$ of each agent a is randomly chosen according to some arbitrary unbiased distribution Φ_a centered around an "environmental" target value $\theta^* \in \mathbb{R}$. We assume an environment that is symmetric with respect to this external state. For example, if x_a signifies the direction of motion then such symmetry implies rotational invariance so that all 360° of motion are initially equivalent and only relative angle measurements are meaningful. Informally, the initial location $x_a(0)$ is perceived as an inaccurate sample of the environment. We typically assume that the variance of Φ_a is know to agent a, also in the more liberal settings we may assume that the full knowledge of the parameterized distribution family $\{(\Phi_a; \theta)\}_{\theta \in \mathbb{R}}$ is available to each agent a. (The family is parameterized by the location of the target $\theta \in \mathbb{R}$).

The goal of each agent a is to relocate itself so that at any given time t, its location $x_a(t)$ would be as close as possible to θ^*. Convergence to the desired value θ^* is achieved by both social interactions and environmental cues[1], where in-between such events agents are free to adjust their internal state and modify their location by making a *move*. That is, an agent a adjusts its location $x_a(t)$ by moving, where a move instruction is specified by some distance quantity $\Delta(X)$, shifting the agent from its current location x to location $x + \Delta(X)$. We view $x_a(t)$ as an *estimator* of θ^*. In particular, we require that at any given time t, the location $x_a(t)$ is an unbiased estimator of θ^*.

1.1.1 Communication

We focus on pair-wise interactions which can be either uni- or bi-directional (our results transfer to interactions with a larger number of agents in a straightforward manner). The information transferred in such interactions may contain *passive* signals that are possibly accompanied by *active* signals. Passive information is obtained as agent a measures its relative distance from agent b, that is,

$$\tilde{d}_{ab}(t) = x_b(t) - x_a(t) + \eta,$$

where the additive noise term, η, is chosen from some arbitrary distribution $N(\eta)$ whose variance is known to the agents. Active signals are modeled as messages that expose some part of the internal memory state of agent b to the observing agent a.

[1] In order for the model to include both social interactions and environmental cues, one or more of the agents can be taken to represent the "environment". The initial locations of these agents are chosen according to highly concentrated distributions, Φ_a, around θ^* and remain fixed thereafter. We capture the increased reliability of a receiving direct environmental cues in comparison to social signals by applying a lower measurement noise to the observation of agents representing the environment.

At this point we note, that in contrast to the internal state $y_a(t)$, we do not assume that the absolute location $x_a(t)$ is known to agent a. That is, we view the surrounding as "relative", meaning in particular, that initially, when $x_a(0)$ is sampled from Φ_a, agent a has no information about the value of its own location $x_a(0)$. In particular, the internal state $y_a(0)$ of each agent a contains merely $\mathrm{var}(\Phi_a)$ and is independent of the actual value $x_a(0)$.

1.1.2 Rounds

Execution proceeds in discrete rounds. In each round (or time step), starting at time 0, each agent may first choose to change (or not) its location by moving, and then, if specified in the meeting pattern $\mathcal{P}$ (see next section), it views another specified agent, thus obtaining some information. To summarize, the round structure is the following:

- Perform internal computation;
- Move by some $\Delta(x)$;
- View (or not) another agent b (obtaining passive and possibly active information).

For simplicity, all three operations, that is, internal computations, move, and view are assume to occur instantaneously, that is, in zero time.

1.1.3 Meeting patterns

A finite execution is associated with a *pattern of meetings* $\mathcal{P}$, which specifies the interactions at any round, up to some final round $T_{\mathcal{P}}$. The system is considered as anonymous, that is we do not assume that the agents are aware of who they view. In addition, agents cannot even assume that they will interact with other agents or not, in subsequent rounds. In particular, the pattern of meeting $\mathcal{P}$ is not known to the agents in advance. We note however, that when formulating the lower bound purposes, we will consider the most liberal version of this model (see algorithm **Opt** below), assume that agents have unique identities, and know the pattern $\mathcal{P}$ in advance.

Independent meeting patterns: Given a pattern of meetings $\mathcal{P}$, agent a and time t, we recursively define the set of *relevant* agents of a at time t, denoted by $\mathcal{R}_a(t, \mathcal{P})$. Informally, only agents in $\mathcal{R}_a(t, \mathcal{P})$ are relevant for the move decision made by a at time t. At time zero, we define $\mathcal{R}_a(0, \mathcal{P}) := a$, and at time t, $\mathcal{R}_a(t, \mathcal{P}) := \mathcal{R}_a(t-1, \mathcal{P}) \cup \mathcal{R}(b, t-1, \mathcal{P})$ if a views b at time $t-1$ (otherwise $\mathcal{R}_a(t, \mathcal{P}) := \mathcal{R}_a(t-1, \mathcal{P})$). A meeting pattern $\mathcal{P}$ is called *independent* if whenever some agent a views some other agent b at some time $t \leq T_{\mathcal{P}}$, then $\mathcal{R}_a(t-1, \mathcal{P}) \cap \mathcal{R}(b, t-1, \mathcal{P}) = \emptyset$.

1.1.4 Random variables

Consider an algorithm A. At any given time t the location and internal state of each agent are random variables which we denote by $X_a(t, A)$ and $Y_a(t, A)$, respectively specific assignments to $X_a(t, A)$ and $Y_a(t, A)$ are denoted by $x_a(t, A)$ and $y_a(t, A)$). The value of a random variable $X_a(t, A)$ indicating the location of a is affected by:

1. The environmental value θ^*,
2. The relative distances of the initial locations of agents $b \in \mathcal{R}_a(t, \mathcal{P})$ (governed by the distributions Φ_b) with respect to θ^*,
3. The noises η in the distance samplings,
4. The moves of agents a and $b \in \mathcal{R}_a(t, \mathcal{P})$
5. The instruction rules of algorithm A itself (which, in particular, may be a randomized algorithm and may thus use coin flips). These rules result in, *e.g.*, the previous moves made by agents and the information transferred by communicating active signals.

The memory random variable $Y_a(t, A)$ is affected by Items (2–5) above, but independent of the value of θ^*. In the case where the pattern of meeting (see below) is chosen by a random scheduler [74], the random variables $X_a(t, A)$ and $Y_a(t, A)$ are affected also by the coin flips made by the random scheduler.

2 The optimal algorithm Opt

2.1 Specification of Opt

Our reference for evaluating the performances of algorithms is the *optimal algorithm*, denoted by **Opt**, which is the best algorithm operating under the most liberal assumptions regarding internal memory resources, internal (individual) computation abilities and communication capacities. That is, when considering algorithm **Opt** we assume no restrictions on the communication capacity, the memory capacity and the internal computation abilities of each agent. Being as liberal as possible, we further assume that active communication occurs without any noise[2]. In addition, we assume that each agent a has a unique identity and is aware of the entire pattern of meeting $\mathcal{P}$ in advance.

Each agent a starts by holding in its memory the full knowledge of the parameterized distribution family (Φ_a, θ) (which includes, in particular, the variance of Φ_a). Without loss of generality, we assume that all individual decisions of a are encoded in its memory. Specifically, whenever moving by some quantity $\Delta(x)$ at some time t, the value of $\Delta(x)$ is stored in the memory $Y_a(t+1)$ of the agent. Note that, in the case where algorithm **Opt** is probabilistic, the choice of $\Delta(x)$ may depend not only on $Y_a(t)$ but also on the results of a sequence $r_a(t)$ of coin flips. In this case, the sequence $r_a(t)$ is written in $Y_a(t)$ as well. Throughout the execution, each agent a will store in its memory $Y_a(t)$ all its meetings' information together with all the previous meeting information stored at the agents it has met, and their previous meeting information, etc.. That is, during an interaction at time $t+1$, the viewing agent a stores in its internal variable $Y_a(t+1)$ not only its internal state before the meeting, namely $Y_a(t)$, and the noisy sample $\tilde{d}_{ab(t)}$ of its distance from the second agent b, but also

[2]The reason, that at this stage, we do not assume noise in active communication is that defining noise for the general type of active communication would require specifying the representation of the internal state, and hence would depend on the algorithm's instructions. For the purposes of lower bounds, we do not want to restrict the algorithm, hence we prefer not to assume noise in active communication. Again, we stress that the reliability of active communication does not allow to completely avoid the noise in passive measurements by encoding the location to the internal state, because an agent is unaware of its actual location.

b's identifier and its full memory content as stored in $Y_b(t)$. (This includes b's initial parameterized distribution family (Φ_b, θ) and all measurements agent b previously took). This leads to an internal state $Y_a(t+1)$ composed of a large nested structure which contains all the information that the agent could possibly obtain at that time from the set of relevant agents $\mathcal{R}_a(t, \mathcal{P})$. Note, given the pattern $\mathcal{P}$, this information $Y_a(t)$ subsums the information it would have received at time t, under any other given algorithm.

The goal of each agent a at time t is to locate itself as close as possible to θ^*, that is to minimize $|x_a(t) - \theta^*|$. (recall that agent a is not aware of value of $x_a(t)$.) Algorithm **Opt** must maintain unbiased estimators $X_a(t)$ at any time t, and among all such estimators, it minimizes the mean square error of $X_a(t)$ around θ^* for any given time t. Note that algorithm **Opt** is well-defined, since the moves used for optimizing the variance at time t do not collide with attempts for optimizing the variance for other times. The reason for that is, that the algorithm at each agent a encodes the previous moves of agent a in a's memory Y_a, and thus these moves can later be subtracted by any other agent that takes into account agent a. Since the noise in the distance samples is additive, this means that without loss of generality, we can assume that, at least when algorithm **Opt** is concerned, the moves of agents do not affect later decisions made by the same or other agents.

2.2 Specification of Opt in an independent meeting pattern

First, please note that all proofs in this manuscript do not rely on the identification of an optimal algorithm. In practice, the performances of any algorithm is evaluated by comparing it to the Cramér-Rao bound. For completeness, we specify the optimal algorithm **Opt** for independent meeting patterns.

Under an independent meeting pattern, algorithm **Opt** can be described explicitly. At any time, each agent keeps a *pdf* in its memory (centered at zero), describing the distribution of the relative distance between the target value θ^* and the current location of the agent. At $t = 0$, since the initial distribution Φ_a is unbiased around θ^*, its mirror image, namely $\Phi_a(-x)$, centered at the zero, is the *pdf* describing the relative distance of θ^* from the agent's location. Note that before any interaction occurs, an agent's optimal strategy would be not to move from its initial location, since any move will either make its location a biased estimator of θ^*, or will increase its variance around θ^*.

Assuming a prior distribution regarding oracle location that is much wider than the uncertainty of any agent, one can use Bayesian considerations to calculate the update rule for interactions between independent agents. In this case, when one agent views another, it updates its *pdf* by point-wise multiplication of its current *pdf* with the noise-convoluted and translated (by the distance measurement) *pdf* of the viewed agent. The agent then relocates itself to the mean of the new *pdf*. More specifically, let $f_{a,t}(x)$ (respectively, $f_{b,t}(x)$) denote the *pdf* of agent a (respectively, b) at time t. Consider a third agent c that has no knowledge regarding its distance from θ^*, which now receives the distribution $f_{b,t}(x)$ and a noisy measurement $\tilde{d}_{cb} = d_{cb} + \eta$ of its distance d_{cb} from agent b. Given this new knowledge, the distribution of the relative distance from θ^* to c is described by the following *pdf*:

$$\int f_{b,t}(x + \tilde{d}_{cb} - \eta)\ N(\eta)\ d\eta.$$

Now, assume that at t, instead of agent c, it is agent a that receives the distribution $f_{b,t}(x)$ and a noisy measurement $\tilde{d}_{ab} = d_{ab} + \eta$. Since the meeting pattern is independent, the previous knowledge

of a regarding its distance from θ^* at time t (described by $f_{a,t}(x)$) is independent from the new information it now received from b. Hence, the distribution of θ^* at time $t+1$ is proportionate to

$$g'_{a,b}(x) = \int f_{a,t}(x) \cdot \int f_{b,t}(x + \tilde{d}_{ab} - \eta)\ N(\eta)\ d\eta.$$

The normalized version of $g'_{a,b}(x)$, termed $g_{a,b}(x)$, therefore describes the distribution of θ^* from its location at time t, given the knowledge obtained by a after viewing agent b. To make its location an unbiased estimator of θ^*, the agent then moves to the mean of this distribution. That is, we have

$$x_a(t+1) = x_a(t) + \int g_{a,b}(x)\ xdx.$$

Finally, the new *pdf* of a at time $t+1$, namely, $f_{a,t+1}(x)$, is $g_{a,b}(x)$ shifted by the last move of the agent, specifically,

$$f_{a,t+1}(x) = g_{a,b}(x - \int g_{a,b}(x)\ xdx).$$

Summarizing the aforementioned discussion, we have the following description of algorithm **Opt**.

Algorithm Opt

- **Compute:** $g'_{a,b}(x) = c \cdot f_{a,t}(x) \cdot \int f_{b,t}(x + \tilde{d}_{ab} - \eta)\ N(\eta)\ d\eta$, Where c is a normalization factor.
- **Update opinion:** $x_a(t+1) = x_a(t) + \int g_{a,b}(x)\ xdx$.
- **Update *pdf*:** $f_{a,t+1}(x) = g_{a,b}(x - \int g_{a,b}(x)\ xdx)$.

In the general, under independent conditions, the *pdf*s maintained by algorithm **Opt** will be complex: To begin with, the initial distributions Φ_a may be already complex in terms of their description. Further, as a result of an interaction, calculating the new *pdf*, as well as its new mean, involve complex operations such as integration and convulsion. Note, with time the *pdf*'s become increasingly more intricate.

3 Fisher information and Relative Fisher information

3.1 Fisher information

3.1.1 Fisher information and the Cremér-Rao bound

We consider a multi-variable probability density function (*pdf*) family $\{(\Phi;\theta)\}_{\theta\in\mathbb{R}}$ where θ is a translation parameter [45]. Let $\bar{z} = \{z_1, z_2 \ldots z_k\}$ be a vector of random variables, distributed according to $(\Phi;\theta)$. The Fisher information of $\{(\Phi;\theta)\}_{\theta\in\mathbb{R}}$ with respect to θ is defined as:

$$J_\Phi^\theta = \int \frac{1}{\Phi(\bar{z},\theta)} \left[\frac{d\Phi(\bar{z},\theta)}{d\theta} \right]^2 d\bar{z}\ .$$

Note, that since θ is a translational parameter, Fisher information is both unique (there is no freedom in choosing the parametrization) and independent of θ [45].

The Cremér-Rao inequality bounds from below the variance of the best possible estimator of θ^* which is based on the random vector sample $\bar{z}$ taken from $(\Phi; \theta^*)$, by the reciprocal of the corresponding Fisher information.

Theorem 3.1. *Let $\hat{X}$ be any unbiased estimator of θ^* which is based on a multi-variable sample $\bar{z}$ taken from $(\Phi; \theta^*)$. Then*

$$\mathrm{var}(\hat{X} - \theta^*) \geq \frac{1}{J_\Phi^\theta}.$$

3.1.2 Initial Fisher-deviation

To define the initial Fisher-deviation parameter Δ_0, we first define the *Fisher-deviation* of a single variable distribution Φ centered at θ^*, as

$$\Delta(\Phi) = \mathrm{var}(\Phi) \cdot J_\Phi^\theta \ .$$

(Note that $\Delta(\Phi) \geq 1$ for any unbiased distribution Φ, by the Cramér-Rao bound). Similarly, the Fisher-deviation $\Delta(N)$ of the noise distribution $N(\eta)$ is defined as:

$$\Delta(N) = \mathrm{var}(N(\eta)) \cdot J_{N-\theta}^\theta.$$

The *initial Fisher-deviation* Δ_0 is the supremum of the Fisher-deviations over all the (unbiased) distributions involved, namely, the Φ_a distributions governing the initial locations and the noise distribution $N(\eta)$. Specifically, let

$$\Delta_0' = \sup\{\Delta(\Phi_a) \mid a \text{ is an agent}\},$$

and finally define

$$\Delta_0 = \max\{\Delta_0', \Delta(N)\}.$$

Observe that if the distributions Φ_a and $N(\eta)$ are all Gaussians then $\Delta_0 = 1$.

3.2 Relative Fisher Information

Fix any pattern of meeting $\mathcal{P}$ and any algorithm A. In addition to the noise $N(\eta)$ on the passive communication, we now consider any level of noise on the active communication (although we do not specify this noise explicitly). We start off by defining the notion of relative Fisher information associated to an agent a at a time t operating under algorithm A. This definition will be used to bound from below the variance of the location $X_a(t, A)$ of agent a at time t around the environmental value θ^*.

Recall, we consider a group of agents that apply algorithm A in order to maintain, for each time t, an unbiased estimator $X_a(t, A)$ of the environmental state θ^*. Note that once the location of the environment is chosen to be some θ^*, all agents' initial locations are chosen with respect to θ^*. Hence, informally, since agents have no knowledge regarding their actual location and only obtain

relative distance samples, from their perspective, all scenarios are identical, regardless of the actual location of the target.

Each agent stores in its memory a multi-valued random variable $Y_a(t, A)$ using it to perform its move $\Delta(x)_{a,t+1,A}$ at the beginning of round $t+1$ (see Subsection 1.1). This would yield its new location at time $t+1$, that is, $X_a(t+1, A) = X_a(t, A) + \Delta(x)_{a,t+1,A}$. Note, it is required from the algorithm A that $X_a(t+1, A)$ remains an unbiased estimator of θ^*.

The random variable $Y_a(t, A)$ is distributed according to some distribution f_y that depends on:

- The collection of initial distributions $\{\Phi_b\}_{b \in \mathcal{R}_a(t,\mathcal{P})}$,
- The pattern of meeting $\mathcal{P}$, the agent a and the time t,
- The rule of algorithm A (determining moves and communication),
- The noise distribution $N(\eta)$.

It is important to note that f_y is independent of θ^*, and therefore one cannot construct an estimator for θ^* by relying on f_y alone. On the other hand, f_y contains information regarding the relative distance between the agent and θ^*.

Consider now an (imaginary) external observer a^* that at any given time needs to estimate the actual target value θ^*. The external observer a^* at the beginning of round $t+1$ receives the pair

$$z_{a^*}(t+1, A) := (y_a(t+1, A), x_a(t, A)),$$

where the sample $y_a(t+1, A)$ is the memory of a at the beginning of round $t+1$, and $x_a(t)$ is the actual location of a at time t. Note that the random variables $y_a(t+1, A)$ and $x_a(t)$ may be dependent. In contrast to $y_a(t+1, A)$, the value of $x_a(t)$ depends on θ^*. Hence, $z_{a^*}(t+1, A)$ is distributed according to a *pdf* family $\{(g_{a^*}(t), \theta)\}$ parameterized by θ. Under some smoothness conditions of A and integrable properties of the noise $N(\eta)$, the noise in the active communication, and the initial distributions $\{\Phi_b\}_b$, the Fisher information $J_{g_{a^*}(t)}(\theta)$ is well defined (see [44]).

Based on $z_{a^*}(t+1, A)$, the external observer needs to output an unbiased estimation $\hat{X}_{a^*}(t+1, A)$ of θ^*, that is, we must have:

$$\text{mean}(\hat{X}_{a^*}(t+1, A) - \theta^*) = 0 \ ,$$

where the mean is taken with respect to the distribution of the random multivariable $z_{a^*}(t, A)$ and, in case a^* is probabilistic, also the coin tosses of a^*, used for deciding its output. For example, one possible estimator would be $x_a(t, A) + \Delta(x)'_{a,t,A}$, where $\Delta(x)'_{a,t,A}$ is the move of a at the beginning of round $t+1$ under algorithm A, assuming a had $y_a(t+1, A)$ in its memory. In this case $\hat{X}_{a^*}(t+1, A)$ is simply $X_a(t+1, A)$, and hence unbiased.

Relative Fisher information: We define the *relative Fisher information* of agent a at the beginning of round t as

$$J_a(t, A) := J_{g_{a^*}(t)}(\theta).$$

By the Cramér-rao bound, the variance $\text{var}(\hat{X}_{a^*}(t, A) - \theta^*)$ of any unbiased estimator $\hat{X}_{a^*}(t, A)$ of θ^* used by the external observer a^* at time t is bounded from below by the reciprocal of the relative Fisher information of agent a at that time. That is, we have:

Lemma 3.2. $\mathrm{var}(\hat{X}_{a^*}(t, A) - \theta^*) \geq 1/J_a(t, A)$.

Since $X_a(t, A)$ is one possible such estimator, we obtain the following.

Lemma 3.3. $\mathrm{var}(X_a(t, A)) - \theta^*) \geq 1/J_a(t, A)$.

Lower bounds on the variance of the location of an agent around θ^* can therefore be obtained by bounding from above the corresponding relative Fisher information.

For simplicity of notation, from here onwards, we refer to the relative Fisher information of agent a at time t simply at as the Fisher information of agent a at time t.

4 Upper bounds on the Fisher information $J_a(t, A)$

4.1 Proof for upper bounds

Throughout this section, we fix any algorithm A used by the agents, and any independent pattern of meeting $\mathcal{P}$. In addition to the noise $N(\eta)$ on the passive communication, we assume any level of noise on the active communication. We assume smoothness properties of A and integrable properties of the noises and the initial distributions $\{\Phi_b\}_b$, so that the corresponding Fisher information at all agents and times are defined.

The goal of this section is to prove the following theorem.

Theorem 4.1. *For any any time $t < T_{\mathcal{P}}$, algorithm A, and independent meeting pattern, the Fisher information of agent a satisfies:*

$$J_a(t+1, A) \leq J_a(t, A) + \frac{1}{\frac{1}{J_b(t,A)} + \frac{1}{J_\eta}}.$$

Proof. Our goal is to bound the Fisher information in the family $\{(g_{a^*}(t), \theta)\}$ parameterized by θ. (Recall, $(g_{a^*}(t), \theta)$ is the density function corresponding to the pair $Z_{a^*}(t) = (Y_a(t), X_a(t))$, assuming the location of the environmental value is θ).

Assume that at time t, agent a views agent b. Let us take a closer look at the random multivariate random variable $Y_a(t+1)$ corresponding to the memory of a at the beginning of round $t+1$. Note that the algorithm A may choose to erase or change some of the information the agent obtained. In addition, the algorithm at b may choose to transmit only part of the memory $y_b(t)$ at b, and this transmission of of the active information may further incur noise. Hence, overall, the memory $Y_a(t+1)$ is a function of:

- The random variable $\tilde{D}_{ab}(t) := X_b(t) - X_a(t) + N$, (corresponding to the noisy distance measurement observed by agent a when viewing agent b),
- The memory $Y_a(t)$,
- The memory $Y_b(t)$.

We now aim at calculating the Fisher information with respect to the parameter θ, available to the outside observer agent a^* at time $t+1$. This is the Fisher information with respect to θ, in the pair $Z_{a^*}(t+1) = (Y_a(t+1), X_a(t))$. Since $Y_a(t+1)$ is a function of $\tilde{D}_{ab}(t)$, $Y_a(t)$, and $Y_b(t)$, the Fisher information in $Z_{a^*}(t+1)$ is bounded from above by the Fisher information in the collection of random multi-variable $(X_a(t), \tilde{D}_{ab}, Y_a(t), Y_b(t))$, see [44]. This latter Fisher information is the same as the Fisher information in the random variables $X_a(t), \tilde{X}_b(t), Y_a(t), Y_b(t)$, where $\tilde{X}_b(t) = X_a(t) + \tilde{D}_{ab}(t) = X_b(t) + N$. Our goal therefore is to bound from above the Fisher information J in the random multivariable $(X_a(t), \tilde{X}_b(t), Y_a(t), Y_b(t))$.

Since the meeting pattern is independent, given the location of the environment value θ, the random multivariable $(X_a(t), Y_a(t))$ is independent of the random multivariable in $(\tilde{X}_b(t), Y_b(t))$. The Fisher information $J_a(t+1, A)$ available to agent a^* at time $t+1$ with respect to θ is therefore bounded from above by the Fisher information J_a in the random multivariable $(X_a(t), Y_a(t))$ with respect to θ plus the Fisher information $\tilde{J}_b$ in the random multivariable $(\tilde{X}_b(t), Y_b(t))$ with respect to θ. That is,

$$J_a(t+1, A) \leq J_a + \tilde{J}_b. \tag{1}$$

The term J_a is precisely $J_a(t, A)$, namely, the Fisher information available to agent a^* at time t. Let us now focus on the rightmost term in Equation 1 and calculate $\tilde{J}_b$, namely , the Fisher information in the random variable $(\tilde{X}_b(t), Y_b(t))$ with respect to θ.

Let $f_{\tilde{X}_b(t),Y_b(t)}[(\tilde{x}_b(t), y_b(t)) \mid \theta = \mu]$ denote the distribution of $(\tilde{X}_b(t), Y_b(t))$, given that the target value is μ. Similarly, let $f_{X_b(t),Y_b(t)}[(x_b(t), y_b(t)) \mid \theta = \mu]$ denote the distribution of $(X_b(t), Y_b(t))$, given a target value of μ. Note that $X_b(t) = \tilde{X}_b(t) - N$, where the noise is distributed according to $N(\eta)$. Thus,

$$f_{\tilde{X}_b(t),Y_b(t)}[(\tilde{x}_b(t), y_b(t)) \mid \theta = \mu] = \int f_{X_b(t),Y_b(t)}[\tilde{x}_b(t) - \eta, y_b(t) \mid \theta = \mu] \; N(\eta) \; d\eta. \tag{2}$$

Let $G(x_1, y_b(t) \mid \mu) := f_{X_b,Y_b(t)}[x_1, y_b(t) \mid \theta = \mu]$, where x_1 is a real valued random variable. Observe that the right hand side of Equation 2 is a convolution of G with N, where the convolution occurs with respect to the first random variable in G, namely, x_1.

The Fisher information inequality [44,45] bounds the Fisher information of a convolution, but it applies to single variable distributions. Essentially, the theorem says that if x, y and θ are real values, $K(x-\theta)$, $R(x-\theta)$ and $Q(x-\theta)$ are a parameterized family and $K = R \otimes Q$, then $J(K) \leq 1/(\frac{1}{J(R)} + \frac{1}{J(Q)})$. We would like to have such an inequality for our convolution of G and N, but we cannot directly apply it to our case, since we have a function G with multiple random variables, where only one of them x_1 being convoluted. We rely on the fact that fact that the random variable $y_b(t)$ does not depend on the location of θ. This fact turns out to be sufficient to also overcome the potential complication rising from the fact that given that $\theta = \mu$, the random variable x_1 may depend on the random multi-variable $y_b(t)$. In Subsection 4.2 we prove Lemma 4.2 which extends the Fisher information inequality to our multi-variable (possibly dependent) convolution case, enabling to prove the following inequality:

$$J(f_{\tilde{X}_b(t),Y_b(t)}[(\tilde{x}_b(t), y_b(t)) \mid \theta = \mu]) \leq \frac{1}{\frac{1}{J(f_{X_b(t),Y_b(t)}[\tilde{x}_b(t), y_b(t) \mid \theta = \mu])} + \frac{1}{J_\eta}},$$

where J_η is the Fisher information in the parameterized family $N(\eta - \theta)$ with respect to θ. In other

words, we obtain:

$$J(f_{\tilde{X}_b(t),Y_b(t)}[(\tilde{x}_b(t), y_b(t)) \mid \theta = \mu]) \leq \frac{1}{\frac{1}{J_b(t,A)} + \frac{1}{J_\eta}}. \quad (3)$$

Together with Equation 1, we obtain the recursive inequality for the Fisher information as required by Theorem 4.1. This completes the proof of the theorem. □

4.2 Extending the Fisher inequality

The Fisher information inequality [44] applies for three one variable distribution families $r(z)$, $p_1(x_1)$, and $p_2(x_2)$ parameterized by μ such that r is a convolution of p_1 and p_2, that is, $r(z) = \int p_1(z-t) \cdot p_2(t)dt$. The theorem gives an upper bound of the Fisher information J_r^μ of the family $r(z-\mu)$ (w.r.t., to μ) based on the Fisher information $J_{p_1}^\mu$ and $J_{p_2}^\mu$ of the families $p_1(x_1-\mu)$, and $p_2(x_2-\mu)$, respectively. Specifically, the theorem states that: $(\alpha_1+\alpha_2)^2 J_r^\mu \leq \alpha_1^2 J_{p_1}^\mu + \alpha_2^2 J_{p_2}^\mu$, for any two real numbers α_1 and α_2. This in particular implies that $1/J_r^\mu \geq 1/J_{p_1}^\mu + 1/J_{p_2}^\mu$.

The following lemma extends the Fisher information inequality to the case where the distributions p_1 and r are composed of multiple, not necessarily independent, variables, where the convolution with p_2 takes place over of one of the variables of p_1.

Lemma 4.2. *Let $r(z, \bar{x}_3 \mid \theta)$, $p_1(x_1, \bar{x}_3 \mid \theta)$, and $p_2(x_2 \mid \theta)$ be three pdfs such that z, x_1 and x_2 are real variables, $\bar{x}_3$ is a vector of multiple real valued variables such that*

$$r(z-\theta, \bar{x}_3) = \int p_1(t-\theta, \bar{x}_3) \cdot p_2(z-t)dt.$$

Then

$$\frac{1}{J^\theta_{r(z-\theta,\bar{x}_3)}} \geq \frac{1}{J^\theta_{p_1(x_1-\theta,\bar{x}_3)}} + \frac{1}{J^\theta_{p_2(x_2-\theta)}},$$

where $J^\theta_{f(\cdot)}$ is Fisher information in the parameterized distribution family f with respect to the parameter θ.

Proof. We start by using the definition of r as a convolution over p_2 and the first variable of p_1:

$$r(z-\theta, \bar{x}_3) = \int p_1(t-\theta, \bar{x}_3) \cdot p_2(z-t)dt.$$

We can insert the density function $p(\bar{x}_3)$ to rewrite the right hand side as:

$$\int p_1(t-\theta|\bar{x}_3) \cdot p(\bar{x}_3) \cdot p_2(z-t)dt$$
$$= p(\bar{x}_3) \int p_1(t-\theta|\bar{x}_3) \cdot p_2(z-t)dt.$$

Implying that:

$$r(z-\theta|\bar{x}_3) = \int p_1(t-\theta|\bar{x}_3) \cdot p_2(z-t)dt.$$

We now define the distributions $R(z) = r(z-\theta|\bar{x}_3)$ and $P_1(t) = p_1(t-\theta|\bar{x}_3)$ so that the previous equation becomes

$$R(z) = \int P_1(t) \cdot p_2(z-t)dt.$$

For which we apply the original Lemma by inequality as proved by Stam [44]: and deduce that for any two real numbers α_1 and α_2, we have

$$(\alpha_1+\alpha_2)^2 J^{\mu}_{R(z-\mu)} \leq \alpha_1^2 \cdot J^{\mu}_{P_1(x_1-\mu)} + \alpha_2^2 \cdot J^{\mu}_{p_2(x_2-\mu)}.$$

We now multiply both sides of the equations by $p(\bar{x}_3)$ and integrate over $\bar{x}_3$, to yield:

$$(\alpha_1+\alpha_2)^2 \int J^{\mu}_{R(z-\mu)}\ p(\bar{x}_3)\ d\bar{x}_3 \leq \alpha_1^2 \int J^{\mu}_{P_1(x_1-\mu)}\ p(\bar{x}_3)\ d\bar{x}_3 + \alpha_2^2 \int J^{\mu}_{p_2(x_2)}\ p(\bar{x}_3)\ d\bar{x}_3. \tag{4}$$

Plugging in the definitions for Fisher information and $R(z)$, the integral on the left hand side becomes:

$$\begin{aligned}
\int J^{\mu}_{R(z-\mu)}\ p(\bar{x}_3)d\bar{x}_3 &= \int J^{\mu}_{r(z-\mu-\theta|\bar{x}_3)} p(\bar{x}_3)d\bar{x}_3 \\
&= \int\int \frac{1}{r(z-\mu-\theta|\bar{x}_3)} \left(\frac{dr(z-\mu-\theta,|\bar{x}_3)}{d\mu}\right)^2\ dz\ p(\bar{x}_3)\ d\bar{x}_3 \\
&= \int\int \frac{1}{r(z-\mu-\theta|\bar{x}_3)p(\bar{x}_3)} \left(\frac{d[r(z-\mu-\theta|\bar{x}_3)p(\bar{x}_3)]}{d\mu}\right)^2\ dz\ d\bar{x}_3 \\
&= \int\int \frac{1}{r(z-\mu-\theta,\bar{x}_3)} \left(\frac{dr(z-\mu-\theta,\bar{x}_3)}{d\mu}\right)^2\ dz\ d\bar{x}_3 \\
&= \int\int \frac{1}{r(z-\mu-\theta,\bar{x}_3)} \left(\frac{dr(z-\mu-\theta,\bar{x}_3)}{d\theta}\right)^2\ dz\ d\bar{x}_3 \\
&= \int\int \frac{1}{r(\tilde{z}-\theta,\bar{x}_3)} \left(\frac{dr(\tilde{z}-\theta,\bar{x}_3)}{d\theta}\right)^2\ d\tilde{z}\ d\bar{x}_3 \\
&= J^{\theta}_{r(z-\theta,\bar{x}_3)},
\end{aligned}$$

where we used $\tilde{z} = z-\mu$ and the fact that $\bar{x}_3$ is independent of θ.

Similarly, the integral over the first term on the right hand side of Equation 4 similarly gives $J^{\theta}_{p_1(x_1-\theta,\bar{x}_3)}$. The last term is

$$\int J^{\mu}_{p_2(x_2-\mu)} p(\bar{x}_3)d\bar{x}_3 = J^{\mu}_{p_2(x_2-\mu)} \int p(\bar{x}_3)d\bar{x}_3 = J^{\mu}_{p_2(x_2-\mu)} = J^{\theta}_{p_2(x_2-\theta)},$$

by normalization of the distribution $\bar{x}_3$.

Finally, Equation 4 translates to:

$$(\alpha_1+\alpha_2)^2 J^{\theta}_{r(z-\theta,\bar{x}_3)} \leq \alpha_1^2 \cdot J^{\theta}_{p_1(x_1-\theta,\bar{x}_3)} + \alpha_2^2 \cdot J^{\theta}_{p_2(x_2-\theta)},$$

for any real α_1 and α_2. Setting $\alpha_1 = J^\theta_{p_2(x_2-\theta)}$ and $\alpha_2 = J^\theta_{p_1(x_1-\theta,\bar{x}_3)}$, we obtain

$$\frac{1}{J^\theta_{r(z-\theta,\bar{x}_3)}} \geq \frac{1}{J^\theta_{p_1(x_1-\theta,\bar{x}_3)}} + \frac{1}{J^\theta_{p_2(x_2-\theta)}},$$

as desired. □

5 An almost optimal compressed algorithm

In this section, we prove that under any given independent pattern of meetings, the performances of algorithm **Conf**, as defined in the main text, are near optimal.

5.1 Mean and variance of estimators in algorithm Conf

Our first goal is to prove that that for any agent a and at any given time t, we have (1) algorithm **Conf** preserves an unbiased estimator $X_a(t) := X_a(t, \mathbf{Conf})$ and (2) the confidence $c_a(t)$ at a equals the reciprocal of the current variance of the location of a with respect to θ^*.

Observe first that if the location of u before the interaction is $x_a(t)$ then after the interaction, its location is

$$x_a(t+1) = x_a(t) + \frac{\tilde{d}_{ab}(t) \cdot \hat{c}_b(t)}{c_a(t) + \hat{c}_b(t)} = \frac{x_a(t)c_a(t) + x_b(t)\hat{c}_b(t)}{c_a(t) + \hat{c}_b(t)} + \frac{\eta\hat{c}_b(t)}{c_a(t) + \hat{c}_b(t)}. \quad (5)$$

Recall that the distributions Φ_a are centered around the environmental value θ^* and that the noise $N(\eta)$ is centered around zero. Moreover, observe that at time t, the confidence at each agent a, namely $c_a(t)$, is deterministically defined (if we fix the pattern of meeting). Equation 5 therefore implies the following, by induction.

Claim 5.1. *At any time t and for any agent a, the location $x_a(t)$ serves as an unbiased estimator of θ^*.*

We are now ready to analyze the variance of the estimator $x_a(t)$ around θ^*.

Lemma 5.2. *Consider algorithm **Conf**. At any time $t \leq T_{\mathcal{P}}$ and for any agent a, we have $c_a(t) = 1/\mathrm{var}(x_a(t) - \theta^*)$.*

Proof. The lemma holds for time $t = 0$ by definition of $c_a(0)$. Assume by induction that for any agent a at time t it holds that $c_a(t) = 1/\mathrm{var}(x_a(t) - \theta^*)$ and consider time $t+1$. We now consider an interaction between two agents at time t, in which agent a views agent b. The variance of the

new location of a is:

$$\begin{aligned}
\mathrm{var}(x_a(t+1)-\theta^*) &= \mathrm{var}\left(x_a(t)+\frac{\tilde{d}_{ab}\hat{c}_b(t)}{c_a(t)+\hat{c}_b(t)}-\theta^*\right) = \mathrm{var}\left(\frac{(x_a(t)-\theta^*)c_a(t)+\hat{c}_b(t)(x_b(t)+\eta-\theta^*)}{c_a(t)+\hat{c}_b(t)}\right)\\
&= \frac{c_a^2(t)\cdot\mathrm{var}(x_a(t)-\theta^*)+\hat{c}_b^2(t)\cdot\mathrm{var}(x_b(t)+\eta-\theta^*)}{(c_a(t)+\hat{c}_b(t))^2}\\
&= \frac{c_a^2(t)\cdot\mathrm{var}(x_a(t)-\theta^*)+\hat{c}_b^2(t)\cdot(\mathrm{var}(x_b(t)-\theta^*)+\mathrm{var}(N(\eta)))}{(c_a(t)+\hat{c}_b(t))^2}\\
&= \frac{c_a^2(t)\cdot 1/c_a(t)+\hat{c}_b^2(t)\cdot(1/c_b(t)+\mathrm{var}(N(\eta)))}{(c_a(t)+\hat{c}_b(t))^2} = \frac{c_a^2(t)\cdot 1/c_a(t)+\hat{c}_b^2(t)\cdot 1/\hat{c}_b(t)}{(c_a(t)+\hat{c}_b(t))^2}\\
&= \frac{c_a(t)+\hat{c}_b(t)}{(c_a(t)+\hat{c}_b(t))^2} = \frac{1}{(c_a(t)+\hat{c}_b(t))} = \frac{1}{c_a(t+1)}\ ,
\end{aligned}$$

which proves the induction step. □

5.2 Competitive analysis of Conf

5.2.1 Competitive analysis - definition

To evaluate the performances of a given algorithm, we compare them to those of the best possible algorithm, namely, algorithm **Opt**. Specifically, for a given time t, the variance of an agent a under an algorithm A is defined as

$$\mathrm{var}(a,t,A) = \mathrm{var}(X_a^A(t)-\theta^*).$$

The competitiveness of algorithm A at time t is

$$\Psi(t,A) := \max\left\{\frac{\mathrm{var}(a,t,A)}{\mathrm{var}(a,t,\mathbf{Opt})} \mid a \text{ is an agent}\right\}.$$

Finally, the competitiveness of algorithm A is defined as

$$\Psi(A) := \max\left\{\Psi(A,t) \mid t \le T_{\mathcal{P}}\right\}.$$

5.2.2 The competitiveness of Conf

To evaluate the performances of algorithm **Conf**, we compare them to those of algorithm **Opt** (see Subsections 2 and 5.2.1), for a given (fixed[3]) independent pattern of meeting $\mathcal{P}$. Specifically, our next goal is to prove that under algorithm **Conf**, for any agent a and any time t, the location of a at time t is an unbiased estimator of θ^*. Furthermore, we shall show that the variance of a's location around θ^* is at most a multiplicative factor of the initial Fisher-deviation Δ_0 over its corresponding

[3]In contrast to algorithm **Opt**, we do not assume that agents operating under algorithm **Conf**, know the pattern $\mathcal{P}$ in advance. Indeed, the instructions of that algorithm are local, taking into account only the current confidences of interacting agents and corresponding distance measurement.

variance at that t under algorithm **Opt** (see Subsection 3.1.2 for the definition of Δ_0). That is, we aim to prove that for any agent a and time $t \leq T_{\mathcal{P}}$ the following equation holds.

$$\mathrm{var}(x_a(t, \mathbf{Conf}) - \theta^*) \leq \Delta_0 \cdot \mathrm{var}(x_a(t, \mathbf{Opt}) - \theta^*). \tag{6}$$

In other words, the competitiveness of algorithm **Conf** at any time t is at most the initial Fisher-deviation Δ_0.

Lemma 3.3 gives a bound on the performances of an agent a operating under algorithm **Opt**, with respect to the Fisher information at the agent. Specifically, we have:

$$\mathrm{var}(X_a(t, \mathbf{Opt}) - \theta^*) \geq 1/J_a(t, \mathbf{Opt}).$$

Initially, the Fisher information $J_a(0, \mathbf{Opt})$ at an agent a equals the Fisher information in the parameterized family (Φ_a, θ) with respect to θ. For a given independent pattern of meeting $\mathcal{P}$, Theorem 4.1 gives a recursive rule for calculating the Fisher information $J_a(t, \mathbf{Opt})$, that is, we have:

$$J(a, t+1, \mathbf{Opt}) \leq J_a(t, \mathbf{Opt}) + \frac{1}{\frac{1}{J_b(t, \mathbf{Opt})} + \frac{1}{J_\eta}}.$$

In the next section, we use this inequality to show that, in fact, under algorithm **Conf**, the variance of agent a at time t, is bounded from above as follows:

$$\mathrm{var}(X_a(t, \mathbf{Conf}) - \theta^*) \leq \Delta_0 / J_a(t, \mathbf{Opt}).$$

Proving this equation would establish Equation 6.

We are now ready to analyze the competitiveness of algorithm **Conf**. We first connect the variance of an agent under **Conf** to its Fisher information under algorithm **Opt**. Recall that $J_a(t, \mathbf{Opt})$ stands for the Fisher information of agent a in time t assuming agents execute the optimal algorithm **Opt**. The following lemma states that the confidence at an agent in algorithm **Conf** equals, up to a multiplicity factor of Δ_0, the fisher information of the corresponding imaginary agent at the same time, in algorithm **Opt**.

Lemma 5.3. *At every time $t \leq T_{\mathcal{P}}$, we have $c_a(t) \geq \frac{J_a(t, \mathbf{Opt})}{\Delta_0}$.*

Proof. The lemma holds initially by the definition of Δ_0. Assume by induction that at time t the lemma holds and consider at interaction at time t when agent a views agent b. By the induction hypothesis and the fact that $c_a(t+1) = c_a(t) + \frac{1}{1/c_b(t) + 1/c_N}$, we obtain:

$$c_a(t+1) \geq \frac{J_a(t, \mathbf{Opt})}{\Delta_0} + \frac{1}{\frac{\Delta_0}{J_b(t, \mathbf{Opt})} + 1/c_N} = \frac{J_a(t, \mathbf{Opt})}{\Delta_0} + \frac{1}{\frac{\Delta_0}{J_b(t, \mathbf{Opt})} + \frac{\Delta_0}{\Delta_0 \cdot c_N}},$$

implying that:

$$\Delta_0 \cdot c_a(t+1) \geq J_a(t, \mathbf{Opt}) + \frac{1}{\frac{1}{J_b(t, \mathbf{Opt})} + \frac{1}{\Delta_0 \cdot c_N}},$$

By definition of Δ_0, we have $\Delta_0 \geq J_\eta / c_N$. Hence:

$$\Delta_0 \cdot c_a(t+1) \geq J_a(t, \mathbf{Opt}) + \frac{1}{\frac{1}{J_b(t, \mathbf{Opt})} + \frac{1}{J_\eta}} \geq J_a(t+1, \mathbf{Opt}),$$

where the second inequality holds by Theorem 4.1. This completes the proof of the lemma. □

Lemmas 5.2 and 5.3 together with Lemma 3.3 imply that

$$\mathrm{var}(x_a(t,\mathbf{Conf})-\theta^*)=\frac{1}{c_a(t)}\leq \Delta_0\cdot\frac{1}{J_a(t,\mathbf{Opt})}\leq \Delta_0\cdot \mathrm{var}(x_a(t,\mathbf{Opt})-\theta^*), \tag{7}$$

establishing Equation 6. Hence, we obtain the following bound.

Theorem 5.4. *The competitiveness of algorithm* **Conf** *is* $\Psi(\mathbf{Conf})\leq\Delta_0$.

5.2.3 On the performances of Conf at large times

We now consider the case that the number of interactions per agent are arbitrary large, and show that for large times, the variance of **Conf** becomes arbitrarily close to zero, and that the performances of **Conf** become closer and closer to those of **Opt**.

We consider noise $N(\eta)$ whose variance is $v_\eta := \mathrm{var}(N(\eta))$. Let $C_{\min}$ denote the minimum initial confidence of an agent in a given population. For fixed values of v_η and $C_{\min}$, we consider larger and larger populations (n goes to infinity), and correspondingly, independent patterns of meeting with large and larger depth M_n.

For a given population, let $C_{\min}(t)$ denote the minimum initial confidence of an agent at time t (in particular, $C_{\min}(0)=C_{\min}$).

Claim 5.5. $\lim_{M_n\to\infty} C_{\min}(t)=\infty$.

Proof. When agent a views agent b the gain in confidence for agent a is

$$\frac{1}{1/c_b(t)+v_\eta}\geq \min\{c_b(t),v_\eta\}/2.$$

It follows that $C_{\min}(t)$ increases in a single round by either a multiplicative factor of 3/2 or by an additive factor of $v_\eta/2$. This implies that the confidences go to infinity as time go to infinity. □

For a given population, let $\mathrm{var}_{\max}(t)$ denote the maximal variance of an agent at time t under algorithm **Conf**. Claim 5.5 together with Claim 5.2 immediately imply the following.

Lemma 5.6. $\lim_{M_n\to\infty}\mathrm{var}_{\max}(t)=0$.

Clearly, since algorithm **Opt** is superior over algorithm **Conf**, the same limit property of the variance applies to algorithm **Opt** as well. We now claim that in fact, if the noise $N(\eta)$ is Gaussian, then the variances in **Conf** and **Opt** go to zero at roughly the same speed.

Lemma 5.7. *If the noise* $N(\eta)$ *is Gaussian then* $\lim_{t\to\infty}\Psi(\mathbf{Conf},t)=1$.

Proof. Since the noise is gaussian we have $v_\eta=1/J_\eta$. (Recall, J_η is the Fisher information in the Noise, that is $J_\eta := J_{(N-\theta,\theta)}(\theta)$). Note now that as $c_a(t)$ become larger and larger the gain in confidence becomes very close to J_η. Specifically, we have

$$c_a(t+1)=c_a(t)+\frac{1}{1/c_b(t)+1/J_\eta}.$$

Consider now the case that $c_b(t) > x \cdot J_\eta$, for some large x. Here, the increase in confidence at a is some quantity $\Delta J_{\mathbf{Conf}}(t)$, satisfying

$$\frac{1}{1+1/x} J_\eta \leq \Delta J_{\mathbf{Conf}}(t) \leq J_\eta.$$

The Cremér-Rao bound and Lemma 5.2 imply that $J_b(t, \mathbf{Opt}) \geq c_b(t)$, and hence, $J_b(t, \mathbf{Opt}) > x \cdot J_\eta$. This, together with Theorem 4.1, implies that at time t, the increase $\Delta J_{\mathbf{Opt}}(t)$ in Fisher information of a under algorithm **Opt** is some quantity satisfying

$$\frac{1}{1+1/x} J_\eta \leq \Delta J_{\mathbf{Opt}}(t) \leq J_\eta.$$

Hence $\Delta J_{\mathbf{Conf}}(t)$ and $\Delta J_{\mathbf{Opt}}(t)$ are the same quantity up to a multiplicative factor of $\frac{1}{1+1/x}$. Since, by Claim 5.5, x goes to infinity as M_n goes to infinity, we get that:

$$\lim_{t\to\infty} \Psi(\mathbf{Conf}, t) = \lim_{t\to\infty} \sup_a \left(\frac{\text{var}(a, t, \mathbf{Conf})}{\text{var}(a, t, \mathbf{Opt})} \right) \leq \lim_{t\to\infty} \sup_a \left(\frac{J_a(t, \mathbf{Opt})}{c_a(t)} \right) = 1.$$

This completes the proof of Lemma 5.7. □

6 The Fisher Channel Capacity and convergence times

6.1 The Fisher Channel Capacity

Given the rule stated in Theorem 4.1 (and the given interaction pattern $\mathcal{P}$), one could now recursively upper bound the Fisher information $J_a(t, A)$. It is this information which, according to the Cramér-Rao bound, sets a lower bound on the variance of $X_a(t)$ around θ^* (see Lemma 3.3). Theorem 4.1 directly implies the following.

Corollary 6.1.

$$J_a(t+1, A) - J_a(t, A) \leq J_\eta \ .$$

The corollary above sets a bound of J_η for the increase in Fisher information per round. This bound holds with respect to any level of noise in active communication, and in particular, when active communication is noiseless. Note moreover, this bound of J_η on the information increase holds with respect to any algorithm A. In analogy to Channel Capacity as defined by Shannon [67] we term this upper bound as the *Fisher Channel Capacity.*

6.2 Bounds on the convergences time

The restriction on information flow as given by the Fisher Channel Capacity can be translated into lower bounds for convergence time of algorithm A, *i.e.* the time in takes the population of agents to enter a certain tight window ϵ around θ^*. More formally, recall that the variance of an agent a is defined as

$$\text{var}_a(t, A) = \text{mean}((x_a(t, A) - \theta^*)^2),$$

where the mean is taken over all possible random initial locations and communication noises, as well as, possibly, over all random choices made by the agents themselves. The variance of the population is defined as the average of these variances, that is,

$$\mathrm{var}(t, A) = \mathrm{mean}(\mathrm{var}_a(t, A)),$$

where the mean is taken over all agents a.

Given small $\epsilon > 0$, the convergence time $T(\epsilon)$ is defined as the first time it takes until the variance of the population around θ^* is less than ϵ^2. (Note that $T(\epsilon)$ is a random variable.)

Convergence thus requires that the estimator applied by the typical agent has variance that is on the order of magnitude of ϵ^2. In particular, by the Markov inequality, at least half the number of agents must have variance less than $2\epsilon^2$. By Lemma 3.3, such agent a must have large Fisher information, specifically,

$$J_a(t, A) \geq 1/2\epsilon^2.$$

To get some intuition on the convergence time, assume without loss of generality that the number of agents is odd, and let J_0 denote the median initial relative Fisher information of agents (this is the median over the Fisher information $J_{\Phi_a - \theta} = J_a(0)$), and assume $J_0 \ll 1/\epsilon^2$. By definition, more than a half of the population has initial Fisher information at most J_0. By the Pigeon-hole principle, at least one agent a has initial Fisher information less than J_0 and at time t, its Fisher information is at least $1/2\epsilon^2$. Corollary 6.1 thus implies a bound for the convergence time $T(\epsilon)$.

Lemma 6.2. *Assume that $J_0 \ll 1/\epsilon^2$ for some small $\epsilon > 0$, then the following bound holds.*

$$T(\epsilon) \ \leq \ \frac{\frac{1}{2\epsilon^2} - J_0}{J_\eta} \approx \frac{1}{\epsilon^2 J_\eta}.$$

Let $J_t^{\max}$ denote the maximal Fisher information over the agents at time t. In the case where $J_0^{\max} \ll J_\eta$, one can obtain a tighter upper bound for $T(\epsilon)$. Indeed, Theorem 4.1 implies that the maximum Fisher information grows by at most a factor odorf 2 in each round, that is, any time t, $J_{t+1}^{\max} \leq 2J_t^{\max}$. This implies that the time it takes until $J_t^{\max}$ reaches J_η is at most $\log_2(J_\eta/J_0^{\max})$. From this time, the bound implied by Lemma 6.2 holds. Hence, we obtain the following.

Lemma 6.3. *Assume that $J_0^{\max} \ll J_\eta \ll 1/\epsilon^2$. Then the following bound holds.*

$$T(\epsilon) \ \leq \ \log_2 (J_\eta/J_0^{\max}) + \frac{\frac{1}{2\epsilon^2} - J_\eta}{J_\eta} \ \approx \ \log_2 (J_\eta/J_0^{\max}) + \frac{1}{\epsilon^2 J_\eta}.$$

Finally, we consider the case of *exclusively-pairwise* patterns, in which the pattern of meeting is such that an agent is never (or rarely) viewed by more than a single agent in each round. In exclusively-pairwise patterns, Theorem 4.1 implies that the average Fisher information, denoted J_t^{mean}, does not grow by more than a factor of two in each round. Similarly to Lemma 6.3, we obtain the following.

Lemma 6.4. *Consider an exclusively-pairwise pattern of meeting. Assume that $\tilde{J}_0 \ll J_\eta \ll 1/\epsilon^2$. Then the following bound holds.*

$$T(\epsilon) \ \leq \ \log_2 (J_\eta/J_0^{\mathrm{mean}}) + \frac{\frac{1}{2\epsilon^2} - J_\eta}{J_\eta} \ \approx \ \log_2 (J_\eta/J_0^{\mathrm{mean}}) + \frac{1}{\epsilon^2 J_\eta}.$$

7 No active communication

We now compare the **Conf** algorithm to even simpler algorithms that rely only on passive communication. We show that efficient "passive" algorithms exist in some "uniform" settings or extremely noisy ones. We then turn our attention to even simpler algorithms, which are based on a fixed weighted average rule between the location of the observing agent a and the location estimation of the observed agent b. We restrict our analysis to meeting patterns in which each agent views another agent in each round.

7.1 Passive algorithms

When noise is not extremely high, "passive" algorithms seem to become less efficient as the conditions become increasingly non-uniform. To get some intuition, consider the extreme case, in which all agents but one agent a, are initially drawn from the same distribution Φ whose variance is extremely large (*i.e.,* these agents are distributed almost uniformly in some large domain centered at θ^*). Agent a, on the other hand, is drawn from an extremely concentrated distribution around θ^*, making it "extremely knowledgeable". Further assume a uniform pattern of meeting, where in each round, each agent views another agent, chosen uniformly at random among the population. In such a case, active communication could allow a standard rumor spreading yielding very fast convergence, roughly within $\log n$ rounds, where n is the number of agents. On the other hand, it seems difficult to come up with a "passive" algorithm that competes well with **Conf**, as agents should somehow distinguish the knowledgeable agent a from the rest. For example, clearly, had the passive algorithm been executed with only the unknowledgeable agents (without agent a), it would not have converged fast. The fact that the algorithm must be unbiased and of low variance, would evidently cause, that in such a case, some of the agents would be anyways at the vicinity of θ^*. Now, the agents running in the first scenario, should somehow distinguish the knowledgeable agent a from these other nearby agents that appear similar, in the second scenario. However, this is most likely impossible since agents are anonymous and active communication is not allowed

7.2 Fixed linear combination of locations

We now consider algorithms in which the update rule following an interaction is a simple linear combination of the location of the viewing agent and the estimated location of the viewed agent. More precisely, when agent a views agent b at time t is shifts its location such that:

$$x_a(t+1) = x_a(t) + c \cdot \tilde{d}_{ab} = (1-c) \cdot x_a(t) + c \cdot (x_b(t) + \eta), \tag{8}$$

for some constant $0 < c < 1$ (note that in algorithm **Conf**, c is not constant and is set according to the active message and a's current confidence). Informally, we find that such algorithms compare well with **Conf** under circumstances which are both very uniform and non-noisy. However, for large values of noise or if initial distributions significantly vary, the performances can be far from optimal.

7.2.1 Speed vs. accuracy tradeoff in uniform initial conditions

We focus first on initial conditions in which all agents are picked from distributions around the target θ^* which all have the same variance $\mathrm{var}(0) \gg v_\eta = \mathrm{var}(N(\eta))$. In this case, at a given time t, the variances of all agents are the same, and we term this value by $\mathrm{var}(t)$. According to Equation 8, this variance satisfies the following recursion rule:

$$\mathrm{var}(t+1) = (1 - 2c + 2c^2) \cdot \mathrm{var}(t) + c^2 \cdot v_\eta \ .$$

The solution to this difference equation is:

$$\mathrm{var}(t) = \left(\mathrm{var}(0) - \frac{c \cdot v_\eta}{2(1-c)}\right)(1 - 2c + 2c^2)^t + \frac{c \cdot v_\eta}{2(1-c)} \ .$$

The steady state variance of this equation scales as $c/(1-c)$ and improves as c is smaller. However, for small values of c, the time to reduce the variance by half scales as

$$t_{\frac{1}{2}} = \frac{1}{\log \frac{1}{1-2c+c^2}} \ ,$$

which diverges for small values of c. Linear combination algorithms therefore exhibit a convergence time vs. accuracy tradeoff.

7.2.2 The simple average algorithm

We start with the simplest, most natural, algorithm in which agents simply average their location with the estimated location of the agent they interact with, this is the case $c = \frac{1}{2}$. This algorithm converges within time $T \geq \log(\mathrm{var}(0)/v_\eta)$ to the steady state:

$$\mathrm{var}(\infty) = \frac{v_\eta}{2}.$$

In comparison with algorithm **Conf** whose variance around θ^* reaches arbitrary small values (see Lemma 5.6), the variance of this simple average algorithm never goes below $v_\eta/2$. On the other hand, in the limit in which population variances are well above v_η, the rules of **Conf** reduce to this simple average algorithm. Therefore, for very small variance values of the noise, the performance of this simple average algorithm compares with that of **Conf** in terms of both convergence rate and steady state variance.

7.2.3 Obtaining lower variance at the price of long convergence times

At steady state the Fisher information passing through the channel is 2/3 of the maximal capacity. The simple average algorithm, obviously, takes no advantage of this information since, at steady state, the Fisher information of the agents remains fixed. To lift this restriction we can choose:

$$c = \frac{\frac{1}{\epsilon^2 + v_\eta}}{\frac{1}{\epsilon^2} + \frac{1}{\epsilon^2 + v_\eta}}.$$

Focusing again on uniform meeting pattern and taking ϵ such that $\epsilon^2 \ll v_\eta$, the variance at time $t+1$ can be approximated as:

$$\text{var}(t+1) = \left(1 - \frac{2\epsilon^2}{v_\eta}\right)\text{var}(t) + \epsilon^4 v_\eta.$$

It's easy to see that the steady state of this equation is $\text{var}(\infty) = \epsilon^2$ which we can choose to be arbitrarily small. This, however, comes at the cost of very long convergence times:

$$T \approx \log_{1-\frac{2\epsilon^2}{v_\eta}}(\text{var}(0)/\epsilon^2) \approx \frac{v_\eta}{\epsilon^2}\log(\text{var}(0)/\epsilon^2) = \frac{v_\eta}{\epsilon^2}\ \left(\ \log(\text{var}(0)/v_\eta) + \log(v_\eta/\epsilon^2)\ \right)\ .$$

Where the first term, the time to reach variance $v_\eta/2$ is much longer than with $c = 1/2$ (see above), and the second term the time to reach steady state from $v_\eta/2$ is much longer, for small $\epsilon > 0$ than what is achieved by the **Conf** algorithm running under similar uniform conditions:

$$T_{\textbf{Conf}} \approx \frac{v_\eta - \epsilon^2}{\eta^2}.$$

8 Extensions of Conf to dynamic environments

The main analysis in this paper was for dissemination of information within a constant environment. This assumption allowed us to perform rigorous analysis and aided in the definition of information flows within the population. The algorithm **Conf**, which we have demonstrated to be highly competitive with an optimal algorithm in stationary environments exhibits weaknesses when the environment is dynamic. These, can mainly be attributed to the buildup of overconfident populations that occurs when agents with similar opinions repeatedly interact and enhance each-others confidence. Once environmental conditions shift this opinion may become wrong but the agents may ignore any new information since their confident in the wrong opinion is extremely high.

In this section we demonstrate how algorithm **Conf** may be extended to accommodate for the case of sudden changes in environmental conditions. These extension require some minimal additional complexity in the capabilities of a single agents.

8.1 Algorithm 1: Communicating an extra 'updated' bit

In this extension the agents perform the regular algorithm **Conf** which is supplemented by storing and communicating an extra "updated" bit in their memory. We take this active communication to be noiseless. The "updated" bit is typically set to 0 (an 'outdated agent') and may be set to 1 ('updated agent') when an agent views the environment, or as described below. The environment is modeled by including an extra mobile agent who is always considered to be updated, and whose confidence is constantly high. We assume that interactions with the environment are rare in comparison to the rumor spread time scale, $\log(N)$, where N is the total number of agents. When an agent becomes updated it changes its update bit to 1 and maintains it at this value for $a \cdot \log(N)$ time steps after which the agent resets this bit to 0 and becomes 'refractory and outdated' for $b \cdot \log(N)$ extra time steps. Finally, $(a + b)\log(N)$ time steps after it had first become 'updated' the agent return to its initial, 'outdated', state.

The remaining interaction rules are as follows:

- When outdated agent views another outdated agent, it performs the regular rules as dictated by **Conf**.
- When an outdated agent observes an updated agent it sets his own confidence to be 0 and then performs the regular rules as dictated by **Conf**. If the observing agent is not refractory he switches to the updated state.
- When a refractory outdated agent observes an updated agent it performs regular **Conf** but does not switch to an updated state.
- When an updated agent observes another updated agent it performs regular **Conf**.
- An updated agent ignores any interactions with outdated agents.

An example for the performance of this algorithm is given in SI-Figure 1A.

8.2 Algorithm 2: Correcting interactions for correlations

Contrary to the algorithm presented in the previous subsection, which complemented the rules of **Conf** by additional rules regarding an extra bit, the algorithm presented in this subsection actually modifies the **Conf** location and confidence update rules. This algorithm follows the modifications to the simple inverse-variance weighting, as suggested by Oruc *et al.* [68], to deal with interactions between agents that possess correlated information. Here, the agents take into account the expected correlations between their locations before an interaction to more accurately update their position and confidence after the interaction.

The interaction rules that are used are an extension to those of **Conf**. Specifically, let v_η denote the variance of $N(\eta)$. Upon receiving $c_b(t)$ and $\tilde{d}_{ab}(t)$, agent a which view an agent b where the correlations between their positions is ρ it proceeds as follows:

Algorithm Conf with correlations

- **Compute:** $\hat{c}_b(t) = c_b(t)/(1 + c_b(t) \cdot v_\eta)$.
- **Correct confidences:** $c'_a(t) = c_a(t) - \rho\sqrt{c_a(t)\hat{c}_b(t)}$, $\hat{c}'_b(t) = \hat{c}_b(t) - \rho\sqrt{c_a(t)\hat{c}_b(t)}$
- **Update external state:** $x_a(t+1) = x_a(t) + \frac{\tilde{d}_{ab}(t) \cdot \hat{c}'_b(t)}{c'_a(t) + \hat{c}'_b(t)}$.
- **Update confidence:** $c_a(t+1) = \frac{c_a(t) + \hat{c}_b(t) - 2\rho\sqrt{c_a(t)\hat{c}_b(t)}}{1-\rho^2}$.

We simulated this algorithm on a uniform population. This is since, in a non-uniform population, the correlation between any two agents may be different and this is difficult to keep track of in an anonymous population of agents with limited memory as interests here. In the simulation, we calculated the correlation between the agents one step at a time so that the correct correlations could be applied to the interaction rules at any time step. We find (see SI-Figure 1B) that before

the environment changes and although the agents continuously interact they do not become overconfident. In fact, after a long time in which all agents interacted with each other, the Fisher information at each agent equals to the initial Fisher information in the entire population. This provides strong evidence that the algorithm suggested by Oruc *et al.* [68] actually performs well within a population setting. Furthermore, the fact that the agents' confidence stays bounded, even at long times, allows them to quickly track an environmental shift when it happens (see SI-Figure 1C).

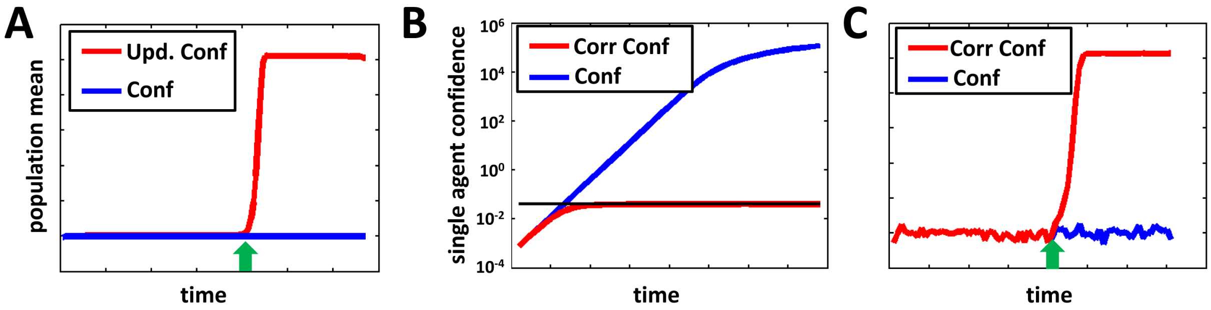

Figure 1: **Extensions of Conf to dynamic environments. A.** In the 'updated bit' algorithm, the agents behave exactly as they would in algorithm **Conf** except for maintaining and communicating a single extra bit which signifies that they have recently received new environmental evidence either directly or indirectly. Agents remain 'updated' for a time of $a \cdot \log(N)$ (N is the number of agents) and then become refractory for another $b \cdot \log(N)$ time steps. The figure shown how this modified **Conf** algorithm follows an environmental change (at green arrow), while **Conf** cannot. Here $a = 2$, $b = 7$ and $N = 10^4$, communication noise is present. **B.** Agents use a modified interaction rule that takes into account the correlation between them prior to the interaction. While under the **Conf** algorithm the confidence of agents is unbounded and explodes (note, that the figure is log scale), this modified algorithm ensures that their confidence remains bounded. In fact the confidence of each agent at long time scale exactly equals the initial Fisher information for the whole population at $t = 0$ (depicted by the black line). This signifies that the agents despite the communication noise the this algorithm allows the agents to share all initial information while remaining realistically confident about their current state. **C.** The fact that under the modified interaction rule the agents do not become over confident allows them to track an environmental shift (green arrow). This, again, stands in contrast to the abilities of the original **Conf** algorithm. In this case, population size was taken to be small ($N = 100$), so that large correlations are created.

9 Heterogeneous populations

In a heterogenic population there is no guarantee that all agents measure distance in exactly the same way. We tested how algorithm **Conf** performs in a population in which agents have a different grasps of distance. SI-Figure 2 shows that the algorithm dominates over an algorithm with no active communication even when different individuals within the population perceive distances with multiplicative errors that vary between $\frac{1}{2}$ and 2. For even larger biases **Conf** still performs better at long but not short time scales.

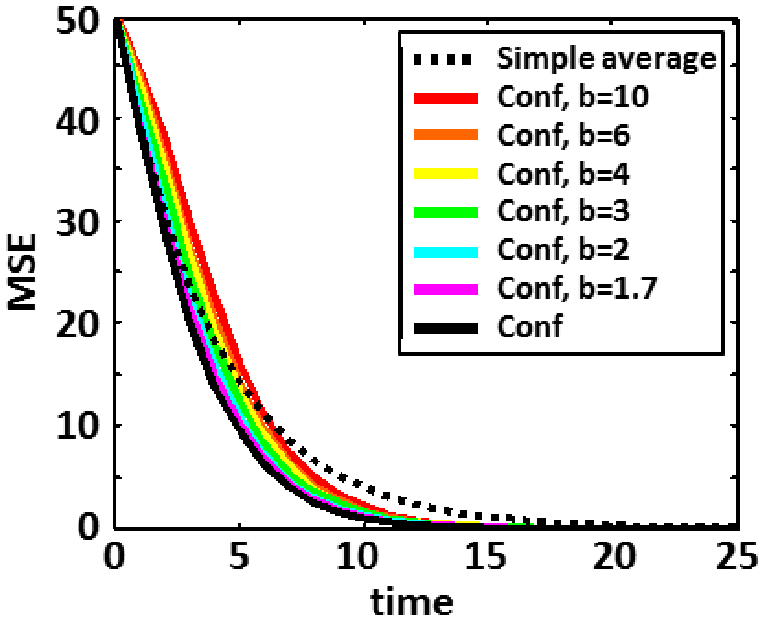

Figure 2: **Algorithm Conf in heterogenic populations.** The convergence of **Conf** on a population of $N = 10^4$ agents that vary in the way they measure distance. To simulate this population each agent was matched with a factor which multiplies all its distance measurements. The factors where chosen uniformly at random in a range that varies with line color. For example, in the population corresponding to $b = 4$ agents perceive distances to be between 4 times smaller or 4 times larger than they actually are. Convergence of population with different biases can be compared to the original **Conf** algorithm ($b = 1$) and to a simple average algorithm that does not communicate confidence and which is unaffected by measurement biases.

10 References

[74] R Karp, C Schindelhauer, S. Shenker, and B. Vocking.Randomized rumor spreading. In *Proceedings 41st Annual Symposium on Foundations of Computer Science*, pages 565–574. IEEE Comput. Soc, 2000.
